# Regional and tele-connected impacts of the Tibetan Plateau surface darkening

**DOI:** 10.1038/s41467-022-35672-w

**Published:** 2023-01-03

**Authors:** Shuchang Tang, Anouk Vlug, Shilong Piao, Fei Li, Tao Wang, Gerhard Krinner, Laurent Z. X. Li, Xuhui Wang, Guangjian Wu, Yue Li, Yuan Zhang, Xu Lian, Tandong Yao

**Affiliations:** 1 Sino-French Institute for Earth System Science, College of Urban and Environmental Sciences, Peking University, Beijing, China; 2 Department of Atmospheric and Cryospheric Sciences, University of Innsbruck, Innsbruck, Austria; 3 Institute of Geography, University of Bremen, Bremen, Germany; 4 State Key Laboratory of Tibetan Plateau Earth System, Environment and Resources (TPESER), Institute of Tibetan Plateau Research, Chinese Academy of Sciences, Beijing, China; 5 Institut des Géosciences de l’Environnement, CNRS, Université Grenoble Alpes, Grenoble, France; 6 Laboratoire de Météorologie Dynamique, CNRS, Sorbonne Université, École Normale Supérieure, École Polytechnique, Paris, France; 7 Laboratoire des Sciences du Climat et de l’Environnement, IPSL, CEA-CNRS-UVSQ, Université Paris-Saclay, Gif sur Yvette, France; 8 Institut Pierre-Simon Laplace, Sorbonne Université/CNRS, Paris, France; 9 Department of Earth and Environmental Engineering, Columbia University, New York, USA

**Keywords:** Climate and Earth system modelling, Cryospheric science

## Abstract

Despite knowledge of the presence of the Tibetan Plateau (TP) in reorganizing large-scale atmospheric circulation, it remains unclear how surface albedo darkening over TP will impact local glaciers and remote Asian monsoon systems. Here, we use a coupled land-atmosphere global climate model and a glacier model to address these questions. Under a high-emission scenario, TP surface albedo darkening will increase local temperature by 0.24 K by the end of this century. This warming will strengthen the elevated heat pump of TP, increasing South Asian monsoon precipitation while exacerbating the current “South Flood-North Drought” pattern over East Asia. The albedo darkening-induced climate change also leads to an accompanying TP glacier volume loss of 6.9%, which further increases to 25.2% at the equilibrium, with a notable loss in western TP. Our findings emphasize the importance of land-surface change responses in projecting future water resource availability, with important implications for water management policies.

## Abstract (web summary)

Impacts of Tibetan Plateau darkening remain unclear. Here authors show that darkening under the RCP8.5 scenario will increase South Asian monsoon precipitation and the “South Flood-North Drought” pattern over East Asia, while lead to local glacier loss.

## Introduction

The rise of the Tibetan Plateau (TP), the world’s highest large landmass, has fundamentally changed the climate in Eurasia^1,2^. Due to its high elevation, the land surface of the TP is characterized by a high albedo (reflective glaciers and dry soils)^3^. With global warming, the land surface of the TP has experienced rapid changes over the last decades^4–6^. Notably, warming-induced greening^7^, glacier melting^6^, and seasonal snow cover changes^8^ lead to reduced surface albedo over the TP^9,10^, i.e., a darkening trend. This darkening trend increases the absorbed shortwave radiation and the land surface temperature, which could further accelerate glacier melting and change the regional water balance^8,11^. However, these important regional climatic and glaciological implications of TP darkening have not been investigated or quantified.

In addition to modifying regional climate and glaciers, the darkening of the TP may also generate tele-connected influences on the thermo-hydrology of other regions through atmospheric dynamics^2,12,13^. The towering TP is a mechanical obstacle to atmospheric circulation up to the middle troposphere^14^. It can divert and divide the westerly jet that dominates the climate of this region and its downstream areas^15–17^, and it is also acted as a large atmospheric heat source (AHS) that plays a unique role in the climate of surrounding regions^18,19^. Thus, darkening-induced changes in the energy balance may modulate water vapor and heat transport to the downstream and surrounding areas, in particular the Asian monsoon region, and further impact those areas’ precipitation patterns. Despite the wide knowledge of the presence of TP in reshaping atmospheric circulation^20^, this potential tele-connected impact of the darkening TP on the Asian monsoon system has not been investigated. Considering that the darkening trend of the TP is expected to continue in a warmer future, it is critical to close the knowledge gap on the regional and tele-connected climatic and glaciological impacts of the TP darkening under future warming.

Here, we assess the impact of albedo changes over the TP on the region’s glaciers and the Asian Summer Monsoon (ASM) under a high-emission scenario, using the high-resolution land–atmosphere global climate model (GCM) LMDZ-ORCHIDEE (LMDZOR), which is a component of the Institut Pierre-Simon-Laplace coupled model (IPSL-CM)^21^, and the open global glacier model (OGGM)^22^. Specifically, we first perform two experiments in LMDZOR: the control experiment (CTL), in which TP albedos are prescribed to current values; and the scenario experiment (SCE), in which future albedos are prescribed only on the TP while albedos in other regions are the same as those in CTL. We estimate the local climate impacts of the darkening TP. Then we compare the LMDZOR simulated ASM between SCE and CTL to assess tele-connected climate impacts by TP albedo changes. Finally, we use near-surface air temperature and precipitation simulated by LMDZOR to drive OGGM and performed two paired (transient and equilibrium) simulations to estimate the albedo-induced glacier melt on the centennial scale, and at the equilibrium state, respectively (see Methods).

## Results and discussion

### Future projected albedo changes in the TP and its impact on local climate

**Fig. 1:**
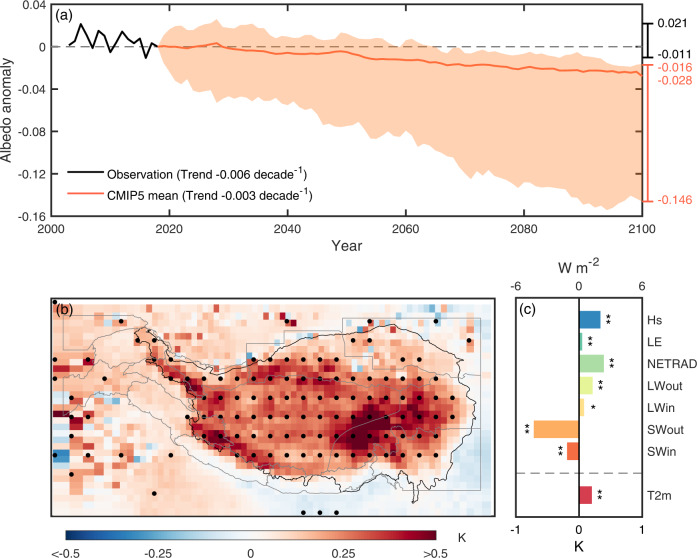
Changes of albedo and albedo-induced local climate in the Tibetan Plateau (TP). **a** Time series of albedo anomaly in the TP. The thick solid black line represents the albedo anomaly of observation during 2003-2018 CE, which is the annual mean albedo derived from the MODIS MCD43 product relative to its benchmark-year (2018 CE) value. The thick solid orange line and shaded orange area, respectively, represent the weighted average and ranges of future (2018-2100 CE) projection albedo anomaly derived from 35 global climate models (GCMs) in CMIP5. The future projection albedo anomaly derived from each CMIP5 GCM is the 5-year moving average of the annual mean albedo in RCP8.5 relative to its benchmark-year (2018 CE) value. Trends of the thick solid black and orange lines are about −0.006 decade^−1^ (*p* = 0.19, Mann-Kendall test) and about −0.003 decade^−1^ (*p* < 0.01, Mann-Kendall test), respectively. **b** The spatial pattern of the albedo-induced near-surface air temperature (t2m) changes. The stippling indicates a significant difference (*p* < 0.05, non-parametric Wilcoxon signed-rank test). The black line indicates the boundary of TP. The gray lines indicate the boundaries of the 13 second-order regions defined by the RGI v6^57,58^. More details about these regions are shown in Supplementary Table 1. **c** The albedo-induced t2m and energy budget-related variables change in the TP. SWin, SWout, LWin, LWout, NETRAD, LE, and Hs, respectively, indicate the downward shortwave radiation, the upward shortwave radiation, the downward longwave radiation, the upward longwave radiation, the net radiation, the latent heat flux, and the sensible heat flux. The symbol “**” indicates the significance at the 99% confidence interval while “*” indicates the significance at the 95% confidence interval. More details about these variables are shown in the Methods.

The future projection of surface albedo used in this study is from a high-emission scenario (RCP8.5) in the framework of the Coupled Model Intercomparison Project Phase 5 (CMIP5) (see Methods). Under the RCP8.5 scenario, a multi-model weighted average of the simulated annual TP albedo at the end of this century (2100 CE) is about 0.028 (10.5%) lower than the benchmark year (2018 CE) value (Fig. 1a and Supplementary Figure 1). Here the model weights are calculated in function of each GCM’s ability in reproducing the spatial variability of observed surface albedo over the TP during the period 2003-2018 CE (see Methods). The substantial shift of TP surface properties is expected to greatly affect the climate system, and is thus investigated with the coupled land-atmosphere global climate model LMDZOR in a paired experiment (CTL: current albedo and SCE: future reduced albedo only over the TP) (see Methods). The albedo-induced climatic effect is then assessed both locally and remotely.

With the decreased surface albedo (Supplementary Fig. 1) under the high-emission scenario, the model simulates a widespread increase of near-surface air temperature over the TP (0.24 ± 0.13 K, regional mean ± standard deviation hereafter, Fig. 1b), with notable warming in the inner TP (0.31 ± 0.08 K) and the southeast TP (0.28 ± 0.17 K). The physical mechanism behind the temperature changes can be understood by examining the variation in different components of the surface energy budget and AHS (see Methods). The surface darkening in the TP under the high-emission scenario induces a significant decrease in the reflected solar radiation (−5.17 ± 3.16 W m^−2^). This decline mainly contributes to an increase in net surface radiation (2.72 ± 1.55 W m^−2^), although it is partly offset by the reduced downwelling shortwave radiation (−1.31 ± 1.88 W m^−2^) and net longwave radiation (−1.15 ± 1.25 W m^−2^, Fig. 1c). The increased net surface radiation is partitioned into sensible (2.48 ± 1.30 W m^−2^) and latent heat flux (0.23 ± 0.77 W m^−2^). Such an increase in net surface radiation, along with the increase of latent heat release due to condensation in some regions such as southeast TP (1.86 ± 1.95 W m^−2^) and Hengduan Shan (1.35 ± 5.28 W m^−2^), significantly contributes to the warming of the TP (0.24 ± 0.13 K) and the enhancement of AHS (2.97 ± 3.59 W m^−2^, 9.9%) (Fig. 1b, c). Compared to the projected total warming of 4.7 K and enhanced AHS by 4.5 W m^−2^ between the end of this century (2080-2100 CE) and the period 2003-2018 CE under the RCP8.5 scenario, the surface darkening over the TP could alone account for an increase of 5.1% for temperature and 66.0% for AHS over the TP (see Methods) (Supplementary Fig. 2a, b).

Projected albedo decline in the TP under a high-emission scenario would also slightly increase precipitation over the eastern TP, such as southeast TP (1.93 mm month^−1^, 2.5%) and Hengduan Shan (1.40 mm month^−1^, 0.6%) while decrease precipitation over the western TP such as Karakoram (−2.27 mm month^−1^, −3.6%) and Hindu Kush (−1.42 mm month^−1^, −4.1%) (Supplementary Fig. 3a). This dipole pattern of precipitation changes over the TP is due to different changes in local convective available potential energy (CAPE, eastern TP: 0.30–1.03 J kg^−1^, 1.0–4.7%; western TP: −0.36 to −0.09 J kg^−1^, −3.4 to −0.8%, Supplementary Fig. 3b) and cloudiness (eastern TP: 3.0–8.6 × 10^−3^, 0.5–1.6%; western TP: −7.9 to −7.3 × 10^−3^, −2.3 to −1.8%, Supplementary Fig. 3c).

### Impact of the TP surface darkening on the ASM

As the TP protrudes into the middle of the troposphere, this plateau-wide surface darkening significantly warms the atmosphere (Fig. 1), which can set up a meridional temperature difference between the TP and oceans that may affect the ASM^19,23,24^.

**Fig. 2:**
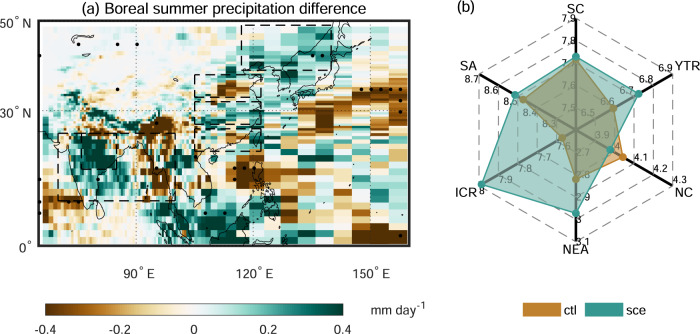
Albedo-induced boreal summer precipitation changes. **a** The spatial pattern of albedo-induced boreal summer precipitation changes in eastern Eurasia. The stippling indicates a significant difference (*p* < 0.05, non-parametric Wilcoxon signed-rank test). The boundaries of six subdivision regions, including South Asia (SA), the Indian subcontinent core monsoon region (ICR), Northeast Asia (NEA), North China (NC), the Yangtze River Valley (YTR), and South China (SC), are highlighted by black dashed lines. The regional averaged boreal summer precipitation in these six subdivision regions is shown in panel (**b**). The unit of the scale in the radar plot is mm day^−1^. More details about these regions are shown in the Methods.

**Fig. 3:**
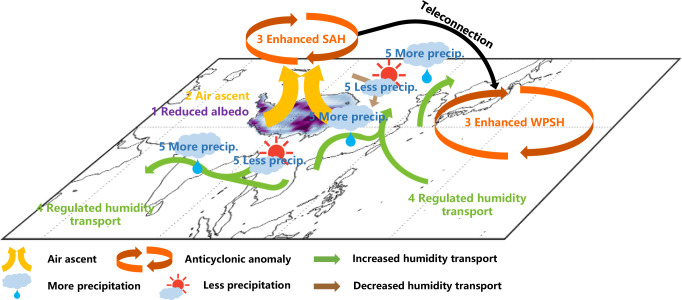
Schematic diagram of the impact of the Tibetan Plateau (TP) surface darkening on the Asian Summer Monsoon. The base map of the figure is derived from Supplementary Fig. 1. The black line indicates the boundary of TP. Reduced albedo in the TP (step one) modulates the local energy budget and thus intensifies the role of TP as an “elevated heat pump”, which ascends more air parcels (step two) and further favors the formation of the atmospheric anticyclonic anomaly (South Asian High, SAH) and the downstream West Pacific Subtropical High (WPSH) through the Rossby wave trains (step three)^33–35^. On the one hand, the intensified anticyclonic anomaly in the troposphere causes the strong easterly wind anomaly over South Asia, which weakens the climatological westerly humidity transport from the Arabian Sea to South Asia, but simultaneously propels moist air from the Bay of Bengal and its surrounding regions to the Indian subcontinent (step four) for the precipitation (“precip.” in the figure, step five). On the other hand, the northwestward shift in the WPSH strengthens the southerly winds in East Asia (step four), which transports more water vapor from the ocean into the interior of the continent for precipitation in Northeast Asia, the Yangtze River Valley, and South China (step five). By contrast, the albedo-induced northerly wind anomaly in North China hinders the moist air from the southern regions triggered by the WPSH (step four), thus conducive to the local dryness (step five).

Results of the equilibrium climate experiments show that, for South Asia, the TP darkening under the high-emission scenario could produce an increase in regional summer monsoon precipitation (1.25 mm month^−1^, 0.5%), especially in the Indian subcontinent core monsoon region (ICR, 12.48 mm month^−1^, 5.5%) (Fig. 2). Such albedo-induced changes in monsoonal precipitation may be originated from the regulation of the atmospheric circulation. The surface darkening enhances the role of the TP as an “elevated heat pump”^25^, favoring the local thermal convection (Supplementary Fig. 4a) and further the formation of an anticyclonic anomaly in the upper troposphere (Supplementary Fig. 4d). The South Asian High (SAH), a characteristic thermal high-pressure system (planetary-scale anticyclonic circulation in the upper troposphere) over the Asian continent in boreal summer^26,27^, then becomes intensified over the TP (Supplementary Fig. 4d), resulting in the strong easterly wind anomaly over South Asia (Supplementary Fig. 4b, c). This albedo-induced easterly wind anomaly weakens the climatological westerly wind and associated humidity transport from the Arabian Sea (Supplementary Fig. 5). But it simultaneously propels moist air from the Bay of Bengal and its surrounding regions to the Indian subcontinent (Supplementary Fig. 5), providing sufficient water vapor in South Asia, especially the ICR, and thus increasing regional monsoonal precipitation (Fig. 3). Note that the precipitation increases in the Indian subcontinent core monsoon region due to the future TP surface darkening (5.5%) are slightly higher than that (4.2%) between the period 2080-2100 CE and 2003-2018 CE under the RCP8.5 scenario (see Methods) (Supplementary Fig. 2c, d).

For East Asia, the TP surface darkening under the high-emission scenario may produce a “north increase-middle decrease-south increase” triple structure of regional summer monsoon precipitation changes. In detail, monsoonal precipitation experiences a decline in North China (NC, −1.97 mm month^−1^, −1.6%), but an increase in its southern regions, including the Yangtze River Valley (YTR, 3.91 mm month^−1^, 2.0%) and South China (SC, 0.35 mm month^−1^, 0.2%), and its northern region, i.e., Northeast Asia (4.57 mm month^−1^, 5.4%). Such darkening-induced triple structure of monsoonal precipitation in East Asia would exacerbate the regional “South Flood-North Drought” (“South” means South China and “North” means North China) pattern^2,28,29^. Specifically, in terms of the difference in boreal summer precipitation between South China and North China, the “South Flood-North Drought” pattern further increases by 19.0% by the end of this century (2080-2100 CE) compared to the period 2003-2018 CE under the RCP8.5 scenario, and nearly one-sixth of this increase is due to the future surface darkening over TP under this scenario (see Methods) (Supplementary Fig. 2e, f).

The triple structure of monsoonal precipitation changes in East Asia due to the projected darkening under the RCP8.5 scenario can be attributed to the strengthening of the SAH and the West Pacific Subtropical High (WPSH) in the troposphere due to the TP darkening (Fig. 3 and Supplementary Fig. 4). The albedo-induced enhancement of SAH could shift the lower-layer WPSH northwestward^30^ through the propagation of a Rossby wave train^31,32^. Specifically, when Rossby waves triggered by the intensification of SAH propagate eastward along the westerly jet, a quasi-barotropic and stationary upper-tropospheric ridge over the coast of China is formed, which further pulls both the SAH and the WPSH, and thus leads to the northwestward (eastward) movement of WPSH (SAH)^33–35^. This northwest-extended WPSH intensifies the low-level southerly winds in East Asia (Supplementary Fig. 4b)^36^ and transports more water vapor from the ocean into the interior of the continent (Supplementary Fig. 5), which enhances the monsoonal precipitation in NEA, the YTR, and SC (Figs. 2 and 3). By contrast, the albedo-induced northerly wind anomaly in NC (Supplementary Fig. 4b, c) would hinder the moist air from the southern regions triggered by the WPSH, thus leading to local dryness (Figs. 2 and 3).

### Impact of the TP surface darkening on the local glaciers

Glaciers, widely distributed in the TP and its surrounding ranges (Supplementary Table 1)^37^, are sensitive to climate conditions^38^. The albedo-induced warming and precipitation changes would therefore impact glaciers over the TP. Using the climate (near-surface air temperature and precipitation) simulated from CTL and SCE in LMDZOR, we perform two transient simulations (called the CTL_t and the SCE_t, respectively) with OGGM^22^, to quantify the response of TP glaciers to the climate on the centennial scale (see Methods).

**Fig. 4:**
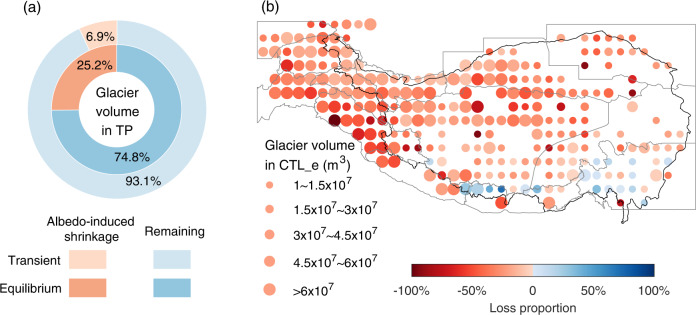
The albedo-induced shrinkage of glacier volume in the Tibetan Plateau (TP) and its surrounding ranges. **a** The proportion of albedo-induced shrinkage of glacier volume and the remaining one on the centennial scale (outer ring, from the transient simulations) and at the equilibrium state (inner ring, from the equilibrium simulations). **b** The spatial pattern of equilibrium-state glacier volume in CTL_e and its loss proportion of SCE_e relative to CTL_e. The loss proportion is computed as the difference between the final 1000-year glacier volume of SCE_e and CTL_e divided by that of CTL_e. The black line indicates the boundary of TP. The gray lines indicate the boundaries of the 13 second-order regions defined by the RGI v6^57,58^. More details about these regions are shown in Supplementary Table 1. The size of each circle represents the equilibrium-state glacier volume at a spatial resolution of 1° in CTL_e, while the color of each circle represents the mean loss proportion of glacier volume in SCE_e relative to CTL_e under the equilibrium state. The glacier volume has been upscaled based on the ratio of the first-year simulations in OGGM and the estimated values from ref. 71 (see Methods).

Results in the transient simulations at the centennial scale show that the surface darkening in the TP under the high-emission scenario induces a glacier loss in terms of both the glacier volume (−6.9%, Fig. 4a) and the glacierized area (−4.6%, Supplementary Fig. 6a) in the final-year simulation of SCE_t relative to that of CTL_t. This albedo-induced difference in the TP glaciers between SCE_t and CTL_t is clearly visible (Supplementary Fig. 7).

Since the glacier response lags behind the changing climate^39,40^, the TP glaciers in both CTL_t and SCE_t do not reach equilibrium by the end of the transient simulations, suggesting that the albedo-induced glacier retreat may continue on a longer time scale. To quantify the impact of albedo changes under the high-emission scenario on the TP glaciers at the equilibrium, a paired 5000-year equilibrium glacier simulation (called the CTL_e and the SCE_e, respectively) is performed (see Methods).

Although the evolution of glacier volume in SCE_e is generally similar to those in CTL_e, the darkening of the TP under the RCP8.5 scenario eventually leads to a lower glacier volume in SCE_e when reaching equilibrium (Fig. 4a). Specifically, compared to the equilibrium stage of glacier volume in CTL_e, 25.2% of glacier volume would disappear in SCE_e (the difference between the simulated final 1000-year average of SCE_e and CTL_e divided by that of CTL_e) of glacier volume will disappear in SCE_e, which is nearly four times as large as the proportion of albedo-induced shrinkage in the transient simulations at the centennial scale (Fig. 4a), and eventually leads to equilibrium loss of the total TP glacier volume by 3.7 × 10^2^ km^3^ (6.1%). In addition, the substantial glacier loss in SCE_e relative to CTL_e can also be reflected in the equilibrium-state glacierized area (−17.9%, Supplementary Fig. 6a). Our results show that the local surface darkening under a high-emission scenario could lead to a more non-negligible reduction of glaciers in the TP and its surrounding ranges on a long-time scale compared with that in the centennial scale.

Both the centennial-scale and equilibrium-state albedo-induced glacier shrinkage varies appreciably across the TP and its surrounding ranges (Fig. 4 and Supplementary Figs. 6 and 7). This highly variable nature of ice losses in space may be attributed to the seasonal and spatial variations in albedo-induced climate change as well as the regional glacier regime. The glacier volume in the southeast TP, in spite of being subject to substantial warming (0.28 K) caused by the darkening of the surface under the high-emission scenario (Fig. 1b), remains relatively stable in both the centennial scale (−2.1%, Supplementary Fig. 7a) and the equilibrium state (−7.0%) (Fig. 4b). This is mainly because the local albedo-induced warming primarily occurs in winter (0.76 K) when the background temperature is still far below 0 °C in SCE of LMDZOR, and increased precipitation in winter (12.7%) and spring (6.8%) favors glacier growth. Similar results can also be found in the south part of the TP (Fig. 4b and Supplementary Fig. 7a), including the east Himalaya (only in the equilibrium state: 6.1%) and the Hengduan Shan (centennial scale: 2.0%, equilibrium state: 0.1%), which may be due to the combination of the slight warming (east Himalaya: 0.03 K, Hengduan Shan: 0.05 K) and the increase in winter (Hengduan Shan: 20.1%) or spring (east Himalaya: 3.0%) precipitation. In contrast, the relatively substantial increase of near-surface air temperature in the west and central part of the TP, including the inner TP (0.31 K), the west Kun Lun (0.23 K), the east Kun Lun (0.21 K), the west Himalaya (0.18 K) and the Karakoram (0.17 K), under the surface darkening conditions of the RCP8.5 scenario would lead to a high glacier volume shrinkage in the corresponding regions, in both the centennial scale (Inner TP: −9.1%, west Kun Lun: −3.1%, east Kun Lun: −5.5%, west Himalaya: −12.6%, Karakoram: −3.5%) and the equilibrium state (Inner TP: −29.4%, west Kun Lun: −26.4%, east Kun Lun: −24.8%, west Himalaya: −50.3%, Karakoram: −24.0%) (Fig. 4b and Supplementary Fig. 7a). Similar substantial shrinkage in glacier volume due to surface darkening also occurs in the southwest and northwest parts of the TP, such as the central Himalaya (centennial scale: −15.5%, equilibrium state: −44.8%), the Hindu Kush (centennial scale: −9.4%, equilibrium state: −35.2%), the Pamir (centennial scale: −9.2%, equilibrium state: −22.7%) and the Hissar Alay (centennial scale: −10.9%, equilibrium state: −22.3%). These regions only experience smaller albedo-induced warming (central Himalaya: 0.05 K, Hindu Kush: 0.13 K, Pamir: 0.12 K, Hissar Alay: 0.11 K) but a substantial decrease in winter (central Himalaya: −15.6%,) or spring (Hindu Kush: −8.1%, Pamir −6.2%, Hissar Alay: −4.5%) precipitation. The only exception is the Qilian Shan, where local darkening conditions cause stable climate changes (near-surface air temperature: 0.08 K, precipitation: 0.1%) but a relatively high retreat in glacier volume (centennial scale: −13.9%, equilibrium state: −29.6%, Fig. 4b and Supplementary Fig. 7a). This may be due to the particular characteristics of the regional glaciers in the Qilian Shan, which are mostly less than 1 km^2^ in size^41^, and show stronger sensitivity to warming compared with larger glaciers^42^.

## Discussion

By using a coupled land-atmosphere global climate model and a glacier model, we show that the decline in projected albedo over the TP (i.e., a darkening trend) under a high-emission scenario could warm the local near-surface atmosphere and strengthen the thermodynamic forcing of the TP. These albedo-induced local changes would hinder the wind and humidity transport over South Asia while augmenting the southerly winds in East Asia, and further redistributing the monsoonal precipitation over the ASM-affected regions. Such tele-connected impacts of the projected surface darkening over the TP on the ASM are weaker than their interannual variabilities (see Methods), but our results indicate shifts in the monsoon mean state, which could be important for the hydrological balance of the surrounding regions. For example, the Indian subcontinent and North China are currently suffering from a summer drying trend, while in South China, summer precipitation is excessive and causes frequent flooding^28,29,43^. These current “South Flood-North Drought” and “West Drought-East Flood” (“West” means South Asia and “East” means South China) patterns may be partly exacerbated (East Asia) or alleviated (South Asia) in our projections with sole decreased albedo over the TP. Note that the current TP surface darkening was not enough to offset the decline in ASM precipitation over the last decades, which is mainly due to increasing anthropogenic aerosols^44^. While, the projected surface darkening over the TP, together with an increase in greenhouse gas emissions and a concomitant reduction in anthropogenic aerosol emissions, would ultimately lead to a revival and enhancement of Asian monsoon precipitation^44,45^. The local and tele-connected climatic impacts of surface darkening over the TP under the future warming scenario are further quantified in our study. In addition, by comparing albedo-induced changes in climate to projected climate changes in the future, our study finds the TP albedo changes would make substantial contributions to the local warming and AHS enhancement, as well as the precipitation increases in the Indian subcontinent core monsoon region and “South Flood-North Drought” pattern in East Asia. This comparison could help isolate the contribution of TP albedo changes to local and remote climate systems in a warmer world.

The darkening of the TP under a high-emission scenario would also lead to glacier retreat over the TP and its surrounding ranges. This albedo-induced glacier retreat is relatively minor though not negligible (area: 4.2 × 10^3^ ± 1.0 × 10^3^ km^2^ or 5.9% ± 2.5%, volume: 4.5 × 10^2^ ± 54 km^3^ or 7.1% ± 1.3%) on the centennial scale (Supplementary Fig. 7), given that the projected glacier loss by the end of this century under the RCP8.5 scenario simulated by ref. 46 is respectively 7.1 × 10^4^ ± 1.3 × 10^4^ km^2^ (area) and 6.2 × 10^3^ ± 1.1 × 10^3^ km^3^ (volume) (see Methods). However, the proportion of glacier loss due to the albedo reduction will further increase over time and becomes more non-negligible in the long term. Such albedo-induced glacier retreat varies considerably across regions, with a high loss proportion in the western TP and a minor one in the eastern TP. But the spatial pattern of such glacier retreat is not always consistent with that of the projected TP albedo decline, suggesting that the impact of albedo on glaciers is not a strict local response, and can also be embodied at a larger scale (e.g., regional scale, Supplementary Fig. 8).

Overall, our findings underscore the critical impacts of the TP darkening on the climate system and hydrological balances in local and remote Asian monsoon regions under a high-emission scenario. Improved quantitative understanding of the climatic and glaciological impacts of the projected TP darkening will help inform policies on alleviating the risk of hydrological-related extremes, better freshwater allocation strategies for both ecosystem and humanity usage, and long-term climatic policies and sustainable development over the TP and in the ASM-affected countries.

## Methods

### Model description

In this study, the climatic effect of the darkening TP was investigated by a coupled land-atmosphere global climate model so-called the LMDZOR, which is a component of the IPSL-CM^21,47^. The model components of LMDZOR used here include the atmospheric module, the Laboratoire de Météorologie Dynamique atmospheric general circulation model with zoomed capability (LMDZ, version 5, v2076), and the land-surface component, the ORganizing Carbon and Hydrology In Dynamics EcosystEms (ORCHIDEE, version 1.9.5, v3035). LMDZ is a state-of-the-art atmospheric general circulation model including relevant dynamical and physical processes governing the atmosphere^48,49^. ORCHIDEE is an advanced terrestrial ecosystem model that simulates surface energy balance and water cycles of soil and vegetation and also considers the terrestrial carbon cycle^50^. In this study, we disabled the carbon cycle module as we primarily focused on the TP albedo effects on the surface energy balance and the water cycle. We intentionally skipped the interactive calculation of land surface albedo by implementing a small module reading the external prescribed shortwave land surface albedo (see Experimental design section below) as boundary conditions. Observational sea-surface temperature (SST) and sea-ice (SIC) were also prescribed as part of the boundary conditions of LMDZOR. An irregular horizontal grid was applied in LMDZOR. Over the TP and the surrounding regions (centered at 33°N, 88°E), spatial resolution reaches about 0.5° × 0.5°. Outside the region of interest, the spatial resolution gradually decreases down to 5° × 1.5° (Supplementary Fig. 9). Such an irregular horizontal grid reconciles an improved simulation of topographic complexity over the TP due to an increase in spatial resolution with the corresponding increase in model instability. However, the spatial resolution of 0.5° × 0.5° can still be too coarse to accurately mimic those TP land surface processes that are strongly influenced by topographic complexity (e.g., ref. 51). At the edge of the zoomed area, LMDZOR can ensure the free exchange of atmospheric water, energy, and momentum. Previous studies have rigorously evaluated and shown the high reliability of LMDZOR in simulating regional climate in East Asia^52,53^. In this study, we further verified the capacity of LMDZOR for simulating the spatial patterns of annual and boreal summer (from May to September) hydrological variables and boreal summer atmospheric circulation over eastern Eurasia and the western Pacific by comparing results in CTL against five different observation-based and reanalysis datasets (see LMDZOR evaluation subsection and Supplementary Figs. 10 and 11 in Supplementary Information).

Here we used the OGGM version 1.5.3 to estimate the effects of surface darkening on glaciers over the TP and the surrounding ranges in this study^22^. OGGM is a modular open-source modeling framework that uses a glacier-centric approach for the simulation of past and future glacier mass balance and its evolution^22^. In this study, the default set-up of OGGM, including the use of elevation band flow-lines^54^; the temperature index model^55^, which was used to calculate the monthly mass balance; the glacier bed inversion (inspired by ref. 56) which was used to estimate the glacier thickness and volume; and the shallow ice approximation^22^ which was used to simulate the glacier dynamics, has largely been followed. The outlines of all glaciers in the TP and its surrounding ranges, which included 13 second-order regions provided by Randolph Glacier Inventory version 6 (RGI v6)^57,58^ (Supplementary Table 1), and the topography of these glaciers obtained from The Global Multi-resolution Terrain Elevation Data 2010 (GMTED2010), were used as the geographical input data to initialize each glacier in OGGM. The parameters of the mass balance model in OGGM have been calibrated through the automated calibration procedure, making use of the geodetic mass-balance data from ref. 59 and pre-processed climatic research unit (CRU) temperature and precipitation^22^. During the CRU climate data pre-processing procedure, the temperature and precipitation derived from the CRU time series (TS)^60^ were downscaled to the CRU climatology (CL)^61^ with the delta method (e.g., ref. 62). This method first computed the deltas of CRU TS relative to its reference climate period (the overlapping part between two climate datasets), then applied these deltas to the CRU CL to obtain the downscaled climate data. The pre-processed CRU climate data would further be used to downscale the climate forcing data for our experiments (see Experimental design section below). A previous study showed that OGGM was successful in exploring future changes in mass balance, volume, and area of glaciers in the Koshi River basin of the TP^63^. We further evaluated the above set-up of OGGM by comparing the simulated glacier changes in the TP and its surrounding ranges over 2000-2016 CE with previous results (see OGGM evaluation subsection and Supplementary Figs. 12–14 in Supplementary Information).

### Experimental design

Two equilibrium experiments were performed using LMDZOR. (I) The control experiment (CTL) is the current albedo experiment, forced by observed climatological surface albedo averaged over a 15-year period of this century (2003-2018 CE) from the Moderate Resolution Imaging Spectroradiometer (MODIS) MCD43 product^64^. The original albedo data at a spatial resolution of 500 m × 500 m was aggregated into 0.5° × 0.5° following ref. 65 to fit the model grid. Other input for CTL in LMDZOR included the average of the same period (2003-2018 CE) observational SST and SIC derived from the input datasets for Model Intercomparison Projects (input4MIPs), fixed global atmospheric CO_2_ data (390 ppm) and the plant functional types (PFT) maps estimated from transient modeling in the project “Trends in net land-atmosphere carbon exchange” (TRENDY), the PFT, and the leaf area index (LAI) obtained from MODIS MCD15 product.

(II) The SCE is the future TP albedo SCE, prescribed with identical lower-boundary conditions (SST and SIC), radiative forcing (CO_2_), and plant dynamics (PFT and LAI) with CTL, but a different albedo map. In contrast to previously perturbed idealized surface albedo experiments, which imposed a spatially-uniform halving of the surface albedo over the TP (e.g., refs. 31, 66), the albedo map used in our simulation considers the spatial heterogeneity in surface albedo changes over the TP in a warmer future. Specifically, we first generated bias-adjusted surface albedo projection from an ensemble of GCMs running under historical conditions (historical, 2003-2005 CE in the study) and a high-emission scenario (RCP8.5, 2006-2100 CE in the study). Among all RCP scenarios, the RCP8.5 scenario agrees most closely (within 1%) with the historical total cumulative CO_2_ emissions during the period from 2005 to 2020 CE^67^, and can therefore be considered a useful scenario to explore climate risks in the future under current policies^67^. Moreover, the use of the highest GHG emission scenario would not lessen the severity of albedo reduction (Supplementary Fig. 15) and associated risk on climate and glaciers, thus keeping the accurate subsequent assessment by governments and the scientific community. Then, the relative changes of the weighted average of multi-model surface albedo between the end of this century (2080-2100 CE) and the period 2003-2018 CE were added to the surface albedo map of CTL, but only over the TP region (Supplementary Fig. 1). We used the Bayesian model averaging (BMA) to assign model weights for each GCM. The BMA is aimed at abating poorly fitting models and elevating high-skilled models through assigning each GCM a weight which is the probability that the model is a “true” model given the observation data^68^, and has been widely used to calibrate the ensembles of climate projection (e.g., refs. 69, 70). We calculated BMA optimal weights for each GCM under the historical and RCP8.5 scenarios based on their ability in reproducing the spatial variability of observed surface albedo over the TP during the period 2003-2018 CE (see details in the Albedo evaluation subsection and Supplementary Fig. 16 in Supplementary Information). Note that the future projection of surface albedo from a high-emission scenario (RCP8.5) used in this study may ignore the consideration of natural variability. The resultant new albedo map was prescribed into LMDZOR to drive SCE.

Both CTL and SCE in LMDZOR were instantaneously imposed of albedo (current and future albedo maps, respectively), and were run for 99 years to enable the simulations to reach an equilibrium state. The last 80 years of the simulations were used for further analysis.

Based on OGGM, we further performed the paired transient (the CTL_t and the SCE_t) and equilibrium (the CTL_e and the SCE_e) simulations, to respectively estimate the centennial-scale and equilibrium-state response of glaciers to the albedo-induced climate changes. All the simulations started from the same initialization but were forced with different climate forcing (near-surface air temperature and precipitation).

In the glacier simulations, the climate forcing, which was used to drive the model, was downscaled by the OGGM pre-processed CRU climate data^60,61^ using the delta method (the period 2003-2018 CE was used as the reference climate period)^22^ (see Model description section above). For CTL_t and CTL_e, the climate forcing was derived from the downscaled near-surface air temperature and precipitation of CTL. The climate data from SCE was also downscaled using the same correction procedure as was applied to the climate data of CTL to drive SCE_t and SCE_e simulations. Thereby, the difference in the climate between CTL and SCE is well preserved in the climate forcing of CTL_t (CTL_e) and SCE_t (SCE_e) simulations. Both transient simulations (CTL_t and SCE_t) were run for 99 years, and only the results from the last year were used for further analysis. For the 5000-year equilibrium simulations, the final 79 years of the pre-processed climate outputs from CTL and SCE were randomly selected 5000 times to generate the TS of the climate forcing to CTL_e and SCE_e, respectively. Only the final 1000-year results of the equilibrium simulations were used for analysis.

### Analysis

Besides the surface albedo, in this study, we also generated the weighted average of CMIP5 GCMs for other climatic variables, including temperature, precipitation, and AHS (Eq. 2) under the historical (2003-2005 CE in this study) and RCP8.5 scenario (2006-2100 CE in the study). The weight of each GCM was the same as that used in generating the weighted average of future TP surface albedo. We further estimated the projected changes of climatic variables between the end of this century (2080-2100 CE) and the period 2003-2018 CE and compared them with the only surface darkening-induced climate change, so as to estimate the contribution of TP surface darkening to future climate change under RCP8.5 scenario. The surface darkening-induced climate change in this study was calculated as the difference of the final 80-year averaged outputs between SCE and CTL. The non-parametric Wilcoxon signed-rank test was further performed for each pair of these 80-year simulations to evaluate whether their difference was significant.

To estimate the albedo-induced glacier changes on the centennial scale, we computed the difference in glacier properties (volume and area) between last year’s results of SCE_t and CTL_t. Similarly, the difference in glacier properties between the final 1000-year-average results of SCE_e and CTL_e was used to represent the albedo-induced changes in glaciers at the equilibrium state. Since some glaciers (contributing to 16% of the total glacier area and 13% of total glacier volume) were not well captured by OGGM, we then upscaled the glacier volume and area through the scaling factor. For glacier volume, the scaling factor is calculated by dividing the first-year simulations in OGGM by the estimated values from ref. 71. For the glacier area, the scaling factor is the ratio of the original glacier area (derived from RGI v6^57,58^) between the well-simulated glaciers and all glaciers. Noted that the glacier volume and area in the TP and its surrounding ranges would ultimately reach the state of dynamic equilibrium, an equilibrium state during which slight fluctuations occur. We also compared the projected glacier loss over Central and South Asia (see refs. 57, 58 and Supplementary Table 1) by the end of this century under the RCP8.5 scenario using the ensemble mean of simulation results conducted by OGGM in ref. 46. For these glacier projections, the OGGM was driven with climate simulations under the RCP8.5 scenario from 10 different GCMs participating in the CMIP5^46^. Assume that the glacier density is 900 kg/m^3^.

The energy budget equation used in this study is:

1 $${{{{{\rm{SW}}}}}}_{{{{{\rm{in}}}}}}-{{{{{\rm{SW}}}}}}_{{out}}+{{{{{\rm{LW}}}}}}_{{{{{\rm{in}}}}}}-{{{{{\rm{LW}}}}}}_{{{{{\rm{out}}}}}}={{{{{\rm{NETRAD}}}}}}={{{{{\rm{Hs}}}}}}+{{{{{\rm{LE}}}}}}+{{G}}$$

where ${{{{{\rm{SW}}}}}}_{{{{{\rm{in}}}}}}$, ${{{{{\rm{SW}}}}}}_{{{{{\rm{out}}}}}}$, ${{{{{\rm{LW}}}}}}_{{{{{\rm{in}}}}}}$, ${{{{{\rm{LW}}}}}}_{{{{{\rm{out}}}}}}$, ${{{{{\rm{NETRAD}}}}}}$, ${{{{{\rm{Hs}}}}}}$, and ${{{{{\rm{LE}}}}}}$, respectively indicate the downward shortwave radiation, the upward shortwave radiation, the downward longwave radiation, the upward longwave radiation, the net radiation, the sensible heat flux, and the latent heat flux. $G$ represents the soil heat flux, and is calculated by the difference among the net radiation, sensible heat flux, and latent heat flux^72^. Since $G$ is relatively small and almost unchanged compared with other terms, its climatological values and albedo-induced changes are not shown in this study.

Here we estimate the annual changes of the AHS over the TP based on the following equation^2,73^:

2 $${{{{{\rm{AHS}}}}}={SH}+{LHR}+{RC}}$$

where ${{{{{\rm{SH}}}}}}$, ${{{{{\rm{LHR}}}}}}$, and ${{{{{\rm{RC}}}}}}$ are the sensible heat flux, the latent heat released due to the change of water phase, and the net radiation flux of the air column, respectively. ${{{{{\rm{LHR}}}}}}$ is estimated based on the following equation^74^:

3 $${{{{{\rm{LHR}}}}}}={\rho }_{w}\times {L}_{w}\times P$$

where ${\rho }_{w}$ indicates the density of water and is equal to 10^3^ kg m^−3^. ${L}_{w}$ = 2.5 × 10^6^ J kg^−1^ is the coefficient of condensation heat and $P$ is the precipitation rate (kg m^−2^ s^−1^). ${RC}$ is calculated as the following equation^73^:

4 $${{{{{\rm{RC}}}}}}={R}_{{{\infty }}}-{R}_{0}=\left({S}_{{{\infty }}}^{\downarrow }-{S}_{{{\infty }}}^{\uparrow }\right)-\left({S}_{0}^{\downarrow }-{S}_{0}^{\uparrow }\right)-\left({F}_{0}^{\downarrow }-{F}_{0}^{\uparrow }\right)-{F}_{{{\infty }}}$$

where ${R}_{\infty }$ and ${R}_{0}$ are the net radiation at the top of the atmosphere (TOA) and the surface, respectively. ${S}_{\infty }^{\uparrow }$ and ${S}_{0}^{\uparrow }$ indicate the upward shortwave radiation at the TOA and the surface, while ${S}_{\infty }^{\downarrow }$ and ${S}_{0}^{\downarrow }$ indicate the downward shortwave radiation at the TOA and the surface. ${F}_{0}^{\downarrow }$, ${F}_{0}^{\uparrow }$, and ${F}_{\infty }$ denote the downward longwave radiation at the surface, the upward longwave radiation at the surface, and the longwave radiation at the TOA, respectively.

We calculated the albedo-induced boreal summer precipitation in six different subdivision regions, including South Asia (10–25°N, 70–100°E), the Indian subcontinent core monsoon region (17–25°N, 76–85°E), Northeast Asia (39–49°N, 117–140°E), North China (33–38°N, 105–122°E), the Yangtze River Valley (27–32°N, 105–122°E), and South China (21–26°N, 105–122°E). Besides, we also computed the standard deviation of the detrended boreal summer monsoon precipitation with a 9-year Gaussian filter^75^ in these six subdivision regions as interannual variabilities of regional boreal summer monsoon precipitation.

## Supplementary information

Supplementary Information

## Data Availability

The OGGM source code can be accessed at https://github.com/OGGM/oggm and the pre-processed directories used in this study can be downloaded at https://cluster.klima.uni-bremen.de/~oggm/gdirs/oggm_v1.4/exps/CRU_new/elev_bands/qc0/pcp2.5/match_geod_pergla/. The projected climate data from the CMIP5 RCP8.5 scenario can be accessed at https://data.ceda.ac.uk/badc/cmip5/data. The observed climatological surface albedo data from the MODIS MCD43 product and the LAI data from the MODIS MCD15 product can be downloaded at https://search.earthdata.nasa.gov/. The observational SST and SIC from input4MIPs are freely available at https://esgf-node.llnl.gov/projects/input4mips/. The global atmospheric CO_2_ data and the PFT maps estimated from TRENDY can be downloaded at https://sites.exeter.ac.uk/trendy. The glacier attribution data from RGI v6 is freely available at http://www.glims.org/RGI/. The elevation data from GMTED2010 can be accessed at https://topotools.cr.usgs.gov/GMTED_viewer/viewer.htm. The CRU TS and CL precipitation and temperature data can be downloaded at https://crudata.uea.ac.uk/cru/data/hrg/ and https://crudata.uea.ac.uk/~timm/grid/CRU_CL_2_0.html, respectively. The consensus estimate for the volume of all glaciers on Earth in ref. 71 is freely available at https://www.research-collection.ethz.ch/handle/20.500.11850/315707, while the future projected glacier changes under the RCP8.5 scenario in ref. 46 can be freely downloaded at 10.1594/PANGAEA.914503. The gridded thickness changes for glaciers in the Himalayans over the period 1975-2016 CE in ref. 76 can be accessed at: https://nsidc.org/data/HMA_Glacier_dH/versions/1, while the ELA of glaciers in the TP and its surrounding ranges is estimated by ref. 77 can be accessed at https://zenodo.org/record/5119153#.Yan0I7q-s2y. The processed output data of climate experiments and glacier simulations generated in this study have been deposited in the “Figshare” (https://figshare.com/articles/dataset/Climate_and_glacier_data/21679409). The boundary of the Tibetan Plateau is derived from https://data.casearth.cn/sdo/detail/5feae826819aec33049b7ca9. Other maps used in this study are derived from MATLAB (see examples: https://ww2.mathworks.cn/help/map/ref/worldmap.html?searchHighlight=worldmap&s_tid=srchtitle_worldmap_1). Other datasets supporting the findings of this manuscript are available in the main text or supplementary information.

## Code availability

The analysis codes used in this study have been deposited in the “Figshare” (https://figshare.com/articles/dataset/Climate_and_glacier_data/21679409).
