# Supplementary material for: Regional and tele-connected impacts of the Tibetan Plateau surface darkening
Source: Nat Commun. 2023 Jan 3;14:32. doi: 10.1038/s41467-022-35672-w (PMC9810690; doi:10.1038/s41467-022-35672-w)
Supplement: Supplementary file 1 — Supplementary Information [file 41467_2022_35672_MOESM1_ESM.docx]

**Regional and tele-connected impacts of the Tibetan Plateau surface darkening**

Shuchang Tang^1^, Anouk Vlug^2,3^, Shilong Piao^1,4^, Fei Li^4^, Tao Wang^4^, Gerhard Krinner^5^, Laurent Z. X. Li^6^, Xuhui Wang^1^, Guangjian Wu^4^, Yue Li^1^, Yuan Zhang^6,7,8^, Xu Lian^1^,

Tandong Yao^4^

^1^Sino-French Institute for Earth System Science, College of Urban and Environmental Sciences, Peking University, Beijing, China.

^2^Department of Atmospheric and Cryospheric Sciences, University of Innsbruck, Innsbruck, Austria.

^3^Institute of Geography, University of Bremen, Bremen, Germany.

^4^State Key Laboratory of Tibetan Plateau Earth System, Resources and Environment, Institute of Tibetan Plateau Research, Chinese Academy of Sciences, Beijing, China.

^5^Institut des Géosciences de l’Environnement, CNRS, Université Grenoble Alpes, Grenoble, France.

^6^Laboratoire de Météorologie Dynamique, CNRS, Sorbonne Université, École Normale Supérieure, École Polytechnique, Paris, France.

^7^Laboratoire des Sciences du Climat et de l'Environnement, IPSL, CEA-CNRS-UVSQ, Université Paris-Saclay, Gif sur Yvette, France.

^8^Institut Pierre-Simon Laplace, Sorbonne Université/CNRS, Paris, France.


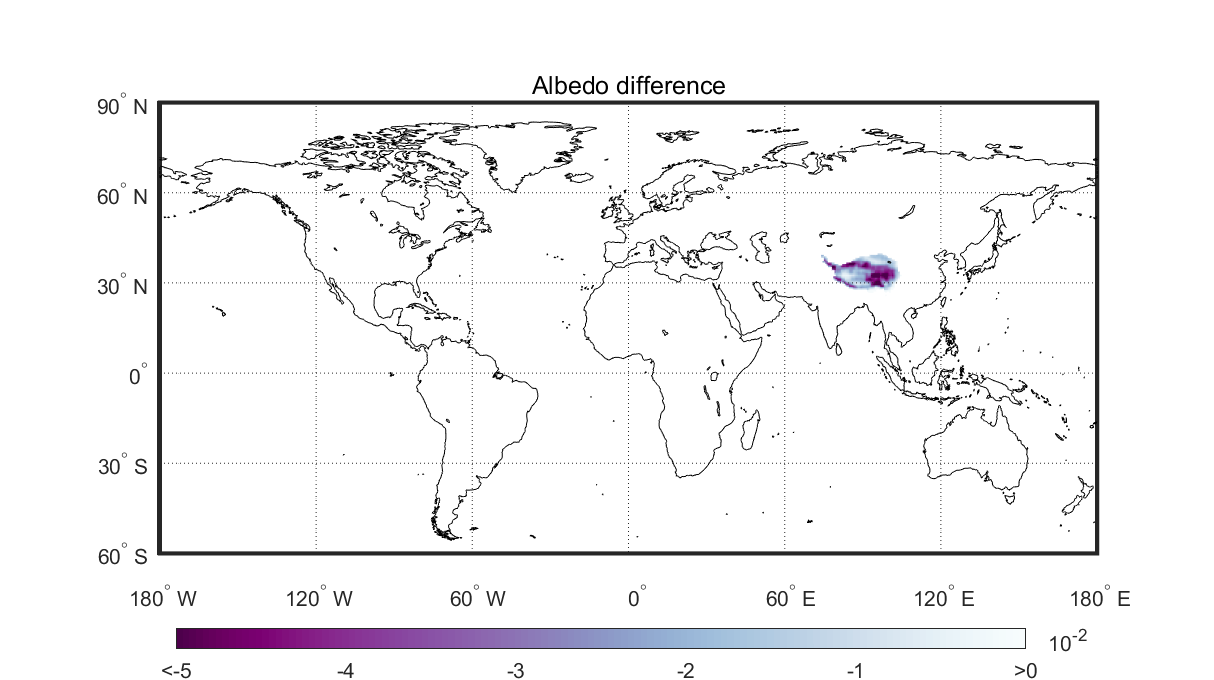


**Supplementary Figure 1.** **The spatial pattern of forced albedo differences between scenario (SCE) and control (CTL) experiments in LMDZOR.** The albedo differences are the changes in the weighted average of surface albedo from CMIP5 global climate models (GCMs) between the end of this century (2080-2100 CE) and the period 2003-2018 CE under the RCP8.5 scenario, and are only in the Tibetan Plateau (TP). More details are shown in the Experimental design section of the Methods, and the Albedo evaluation subsection below.


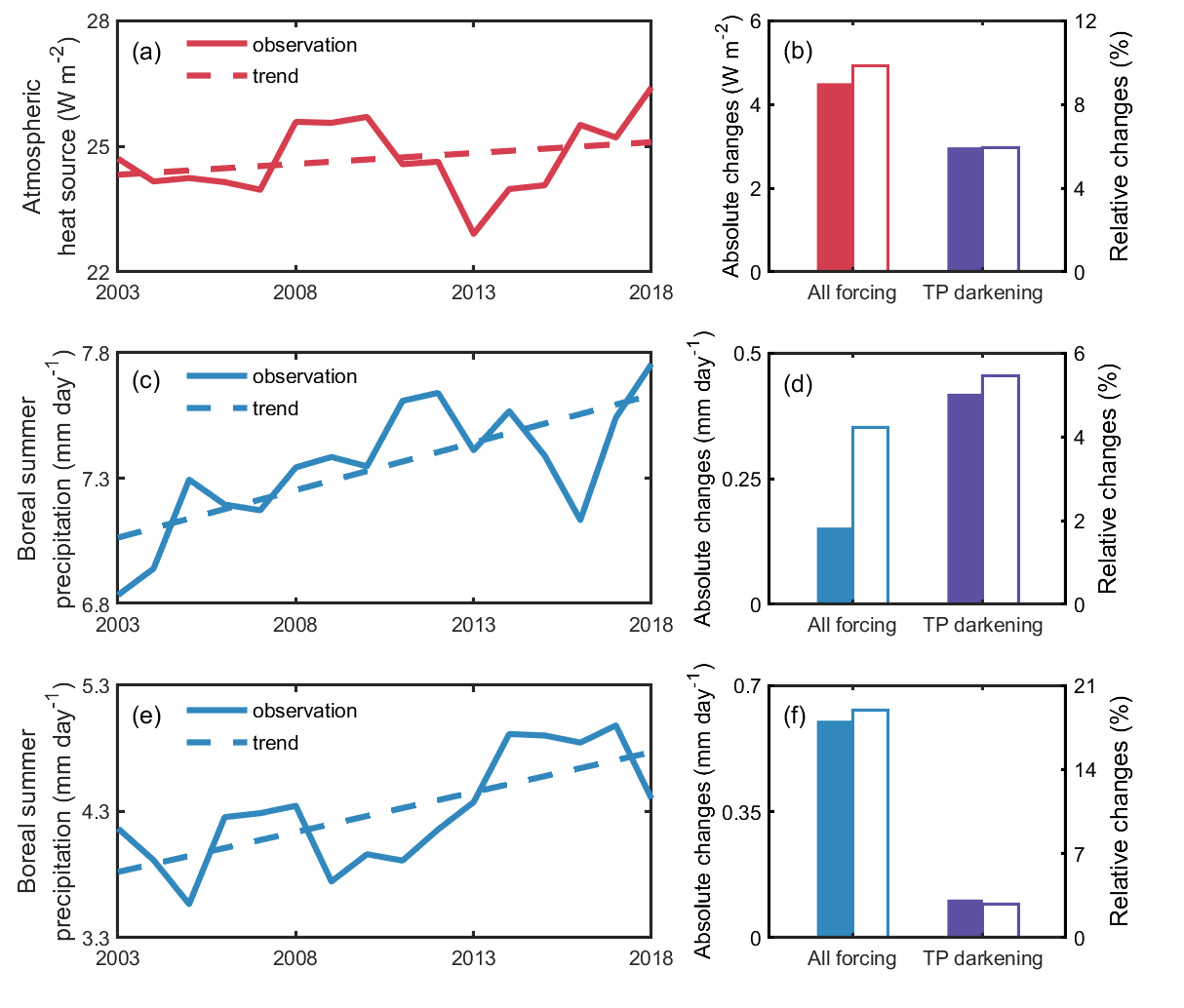


**Supplementary Figure 2. Changes of (a-b) the atmospheric heat source over the Tibetan Plateau (TP), (c-d) the boreal summer precipitation over the Indian subcontinent core monsoon region, and (e-f) its difference between South China and North China.** Panels in the left column are the changes of these variables, including the observation and its trend, during the period 2003-2018 CE. Panels in the right column are the future projected (all forcing, on its left part) and albedo-induced (TP darkening, on its left part) changes of these variables. The future projected changes indicate the difference in the weighted average of CMIP5 global climate models (GCMs) projection between the end of this century (2080-2100 CE) and the period 2003-2018 CE under the RCP8.5 scenario, while the albedo-induced changes are calculated as the difference of the final 80-year averaged outputs between SCE and CTL (see Methods). The filled and unfilled bars indicate absolute and relative changes, respectively. The relative changes refer to the absolute changes relative to the background climatology.


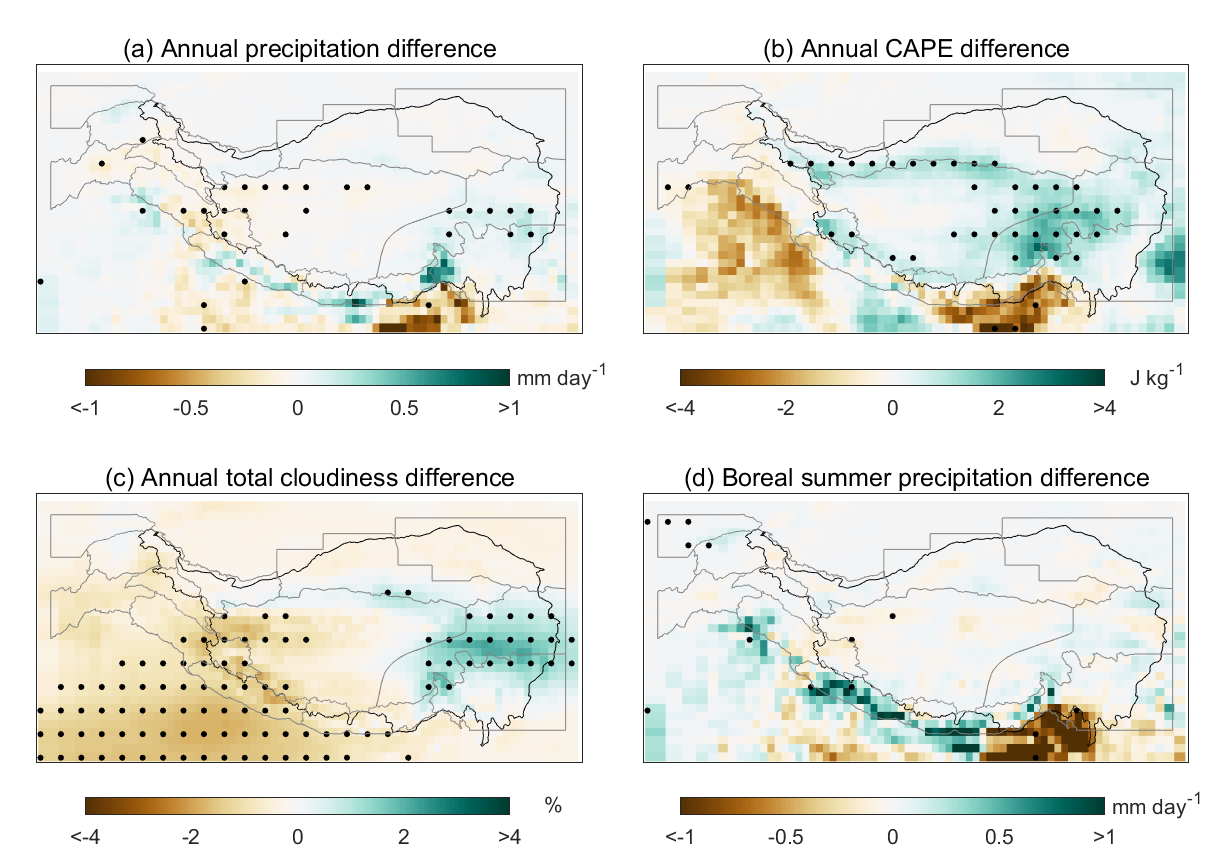


**Supplementary Figure 3.** **Spatial patterns of albedo-induced changes of (a) annual precipitation, (b) annual mean convective available potential energy (CAPE), (c) annual mean cloudiness, and (d) boreal summer (from May to September) precipitation in the Tibetan Plateau (TP) and its surrounding ranges.** The black line indicates the boundary of TP. The grey lines indicate the boundaries of the 13 second-order regions defined by the Randolph Glacier Inventory version 6 (RGI v6) ^1, 2^. More details about these regions are shown in Supplementary Table 1. The stippling indicates a significant difference (p<0.05, non-parametric Wilcoxon signed-rank test).

**
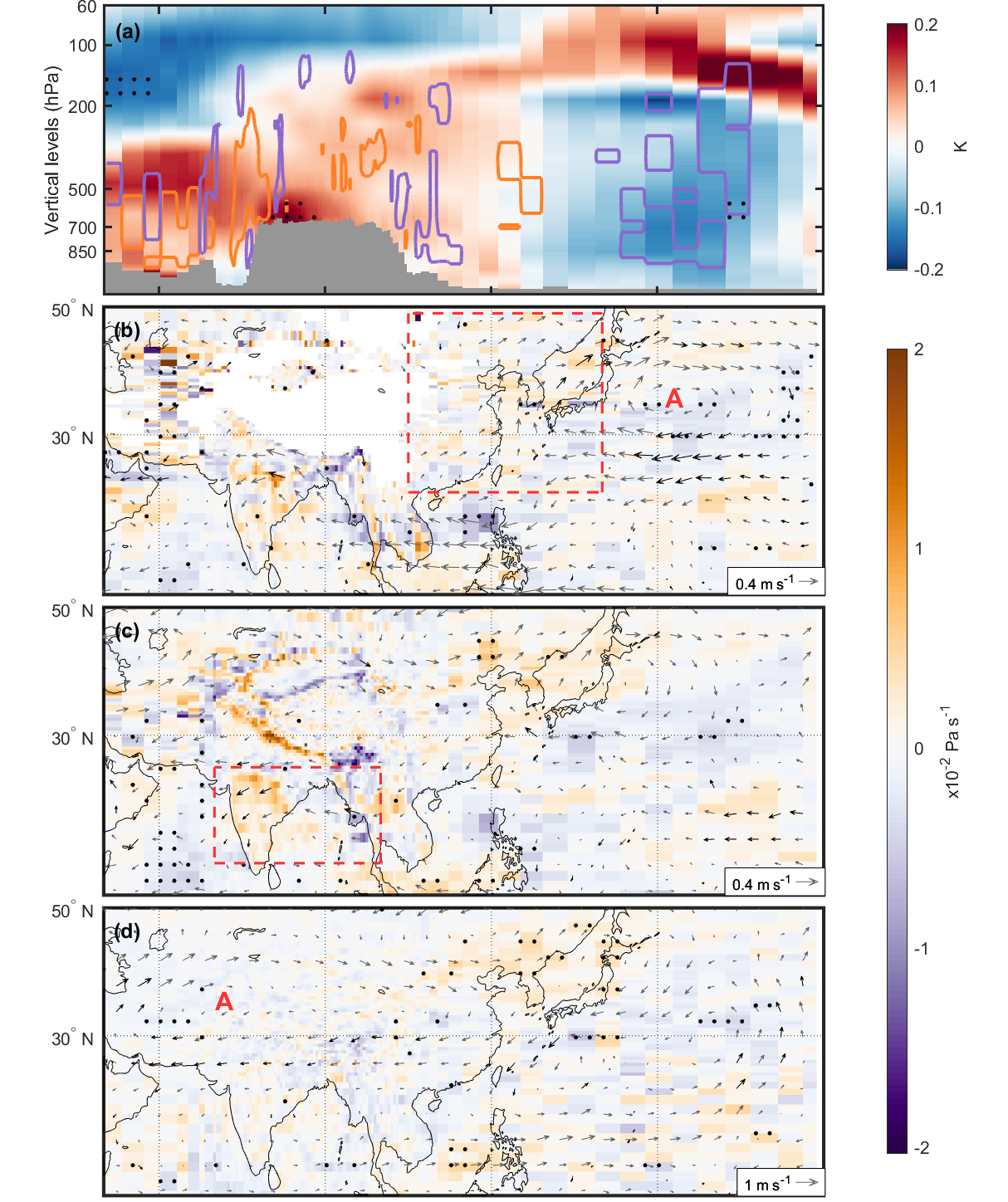
**

**Supplementary Figure 4.** **The albedo-induced boreal summer (a) air temperature and vertical wind changes along the 32.5°N, and spatial patterns of albedo-induced horizontal and vertical winds changes at (b) 850hPa, (c) 500hPa, and (d) 200hPa.** The stippling and black arrow indicate a significant difference (p<0.05, non-parametric Wilcoxon signed-rank test). In panel a, regions without the air temperature (nan value due to the topography) are regarded as the land and masked as grey. The orange (purple) contours are the regions where the upward (downward) vertical winds are larger than 2.5 (lower than -2.5) ×10^-3^ Pa s^-1^. In panels b and d, the symbol “A” indicates the center of the anticyclone circulation anomaly. The boundaries of East Asia (21-49°N, 105-140°E) and South Asia (10-25°N, 70-100°E) are highlighted by red dashed lines in panels b and c, respectively.

**
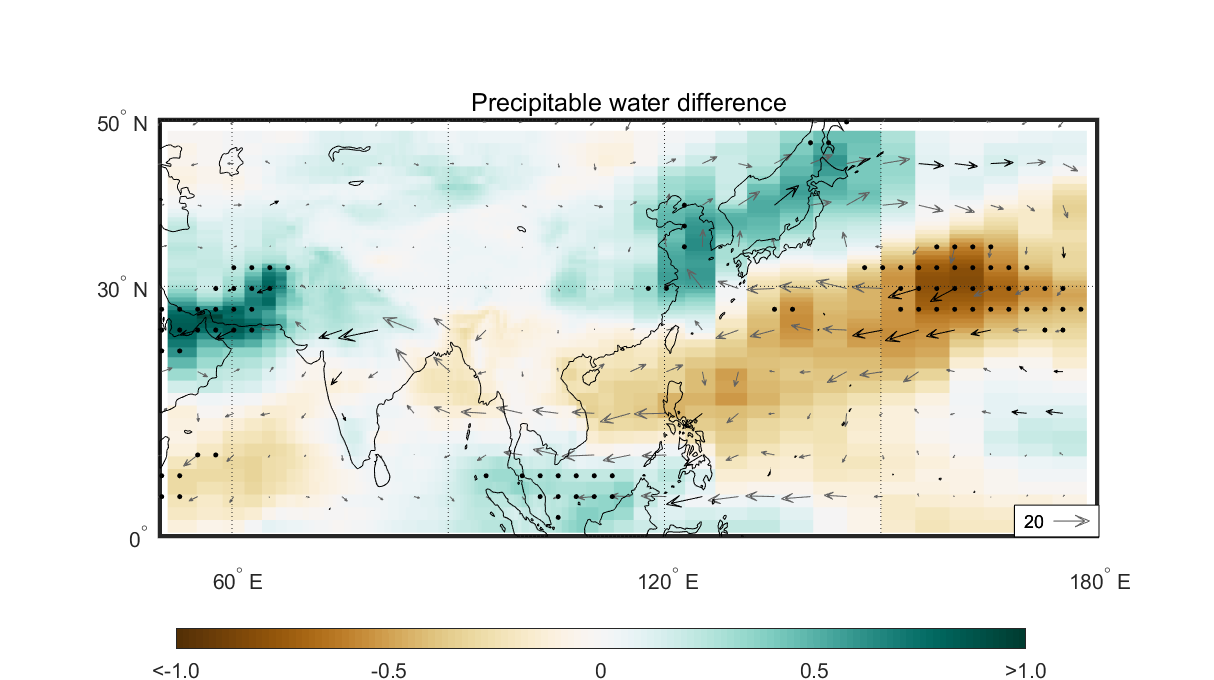
**

**Supplementary Figure 5. The spatial pattern of albedo-induced boreal summer humidity transport (arrow) and precipitable water in the whole atmosphere (shaded) changes.** The stippling and black arrow indicate a significant difference (p<0.05, non-parametric Wilcoxon signed-rank test).

| First-order region | Second-order region | Number of glaciers | Total number of glaciers in second-order regions | Glacierized area | Total glacierized area in second-order regions |
| --- | --- | --- | --- | --- | --- |
| Central Asia (13) | Hissar Alay (13-01)  Pamir (Safed Khirs/W Tarim) (13-02)  W Kun Lun (13-05)  E Kun Lun (Altyn Tagh) (13-06)  Qilian Shan (13-07)  Inner Tibet (13-08)  S and E Tibet (13-09) | 3,147  10,158  5,338  3,502  2,720  9,320  5,042 | 39,227 | 1,846 km^2^  10,234 km^2^  8,153 km^2^  3,251 km^2^  1,637 km^2^  7,923 km^2^  3,873 km^2^ | 36,917 km^2^ |
| South Asia (West) (14) | Hindu Kush (14-01)  Karakoram (14-02)  W Himalaya (14-03) | 4,316  13,686  9,685 | 27,687 | 2,938 km^2^  22,862 km^2^  7,768 km^2^ | 33,568 km^2^ |
| South Asia (East) (15) | C Himalaya (15-01)  E Himalaya (15-02)  Hengduan Shan (15-03) | 4,454  4,094  4,286 | 12,836 | 5,447 km^2^  4,904 km^2^  4,383 km^2^ | 14,734 km^2^ |

**Supplementary Table 1. Details about the 13 second-order regions (defined by the RGI v6, see refs ^1, 2^) in the Tibetan Plateau (TP) and its surrounding ranges for the glacier analysis in this study.**

**
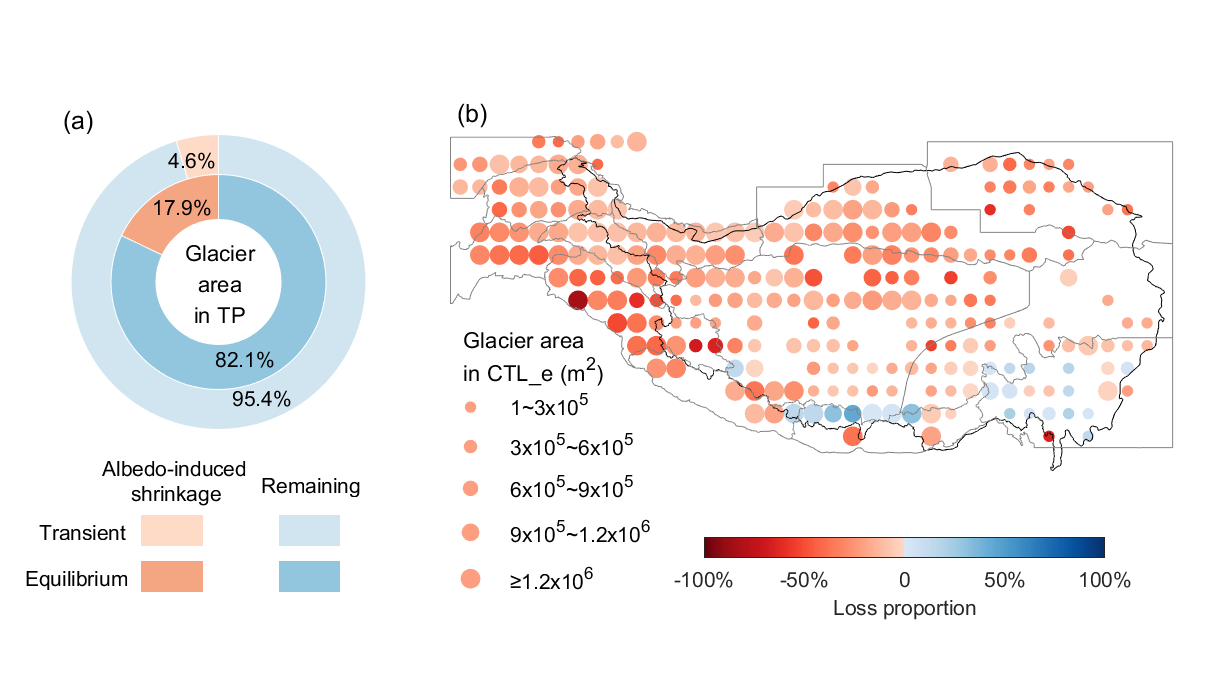
**

**Supplementary Figure 6. The albedo-induced shrinkage of glacier area in the Tibetan Plateau (TP) and its surrounding ranges.** (a) The proportion of albedo-induced shrinkage of glacier area and the remaining one on the centennial scale (outer ring, from the transient simulations) and at the equilibrium state (inner ring, from the equilibrium simulations). (b) The spatial pattern of equilibrium-state glacier area in CTL_e and its loss proportion of SCE_e relative to CTL_e. The loss proportion is computed as the difference between the final 1000-year glacier area of SCE_e and CTL_e divided by that of CTL_e. The black line indicates the boundary of TP. The grey lines indicate the boundaries of the 13 second-order regions defined by the RGI v6 ^1, 2^. More details about these regions are shown in Supplementary Table 1. The size of each circle represents the equilibrium-state glacier area at a spatial resolution of 1° in CTL_e, while the color of each circle represents the mean loss proportion of glacier area in SCE_e relative to CTL_e under the equilibrium state. The glacier area has been upscaled based on the ratio of the original glacier area (derived from RGI v6 ^1, 2^) between the well-simulated glaciers and all glaciers (see Methods).


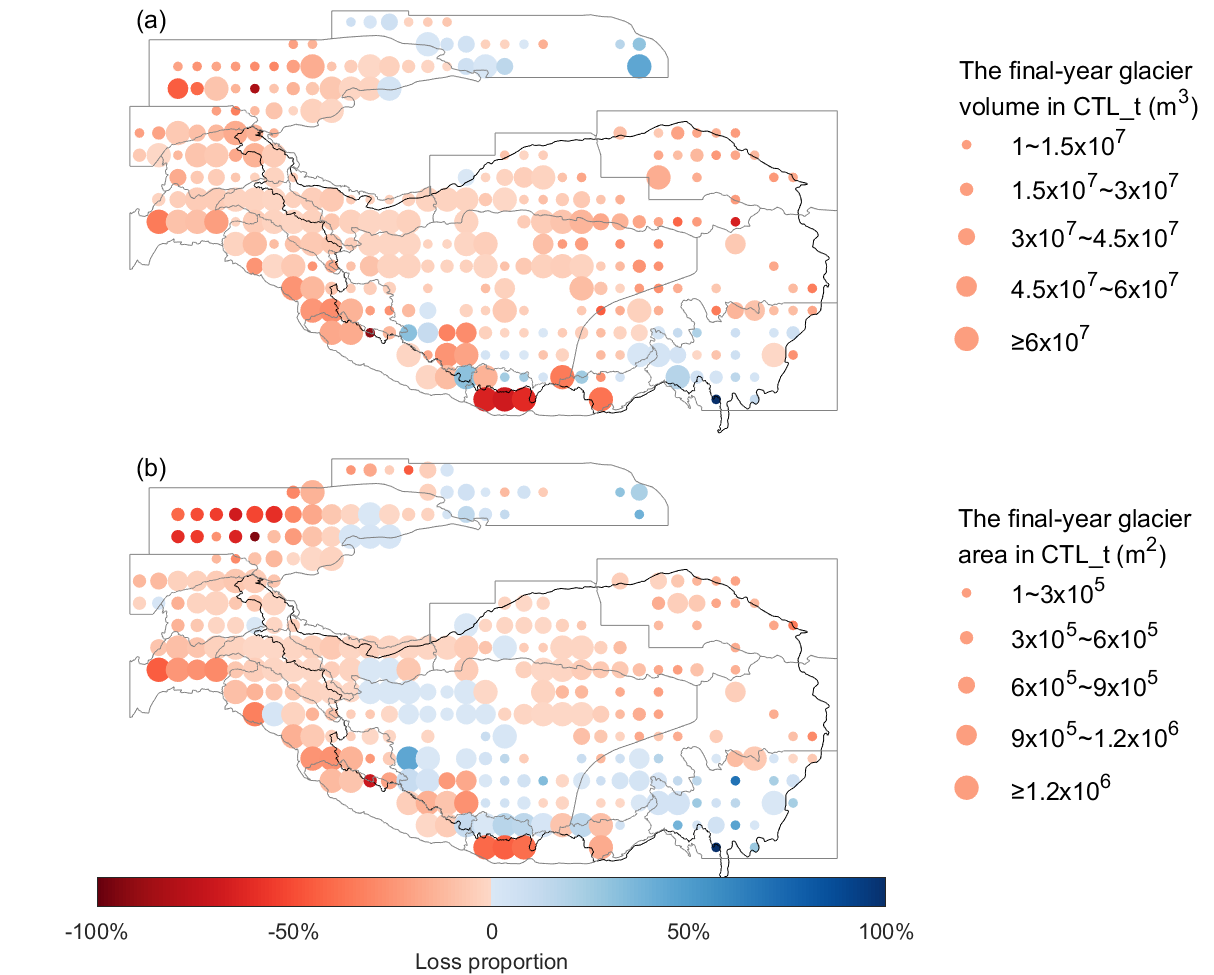


**Supplementary Figure 7. The centennial-scale shrinkage of glacier (a) volume and (b) area over Central and South Asia due to the surface darkening.** The grey lines indicate the boundaries of the 15 second-order regions over Central and South Asia (first-order region 13-15) defined by the RGI v6 ^1, 2^. The black line indicates the boundary of TP. The size of each circle represents the final-year glacier properties (volume or area) at a spatial resolution of 1° in CTL_t, while the color of each circle represents the mean loss proportion of glacier properties. The loss proportion is computed as the difference between the final-year glacier properties of SCE_t and CTL_t divided by those of CTL_t. The glacier volume has been upscaled based on the ratio of the first-year simulations in OGGM and the estimated values from ref ^3^, while the glacier area has been upscaled based on the ratio of the original glacier area (derived from RGI v6 ^1, 2^) between the well-simulated glaciers and all glaciers (see Methods).

**
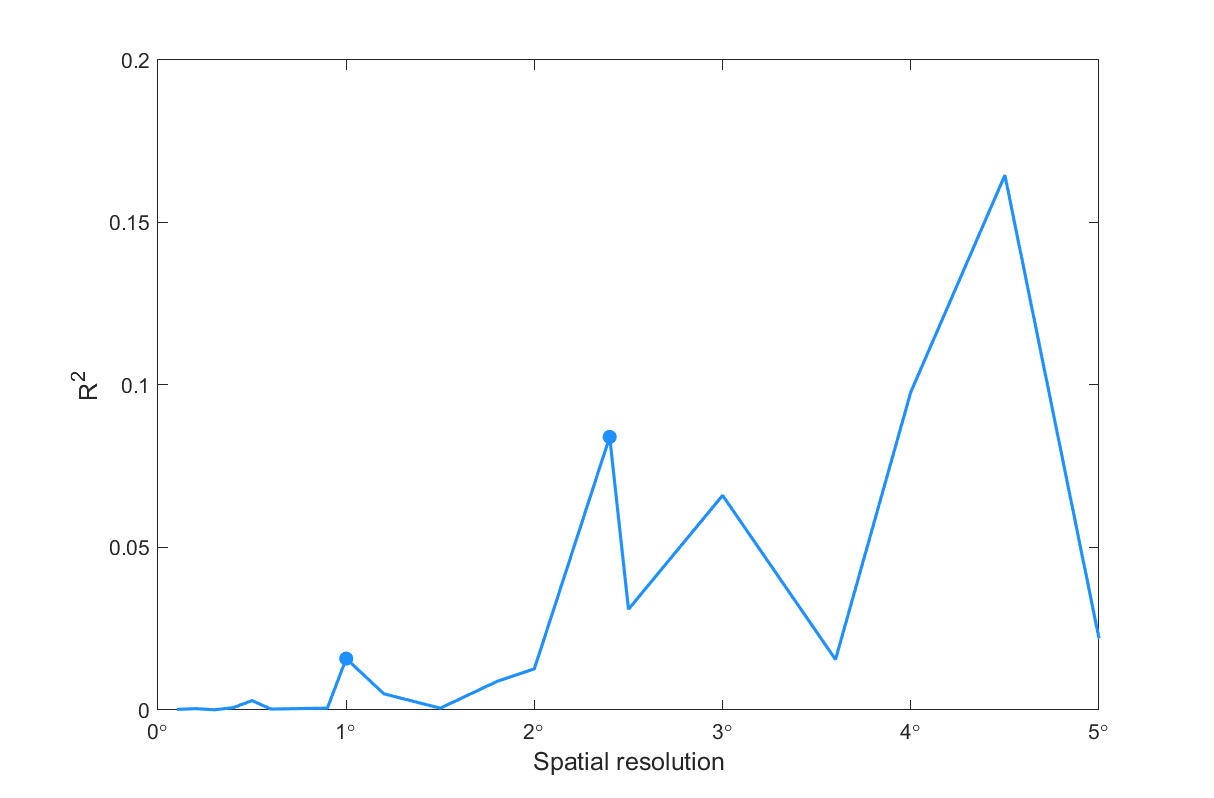
**

**Supplementary Figure 8. Relationship between gridded albedo changes and equilibrium glacier volume loss proportion in the Tibetan Plateau (TP) and its surrounding ranges under different spatial resolutions.** Only grid points with glaciers are selected for analysis. The relationship here means the R^2^ statistic of the linear regression between gridded albedo changes and equilibrium glacier volume loss proportion. The loss proportion is computed as the difference between the final 1000-year glacier volume of SCE_e and CTL_e divided by that of CTL_e. The dots here mean the relationship at this specific spatial resolution is significant (p<0.05, Student’s t test).


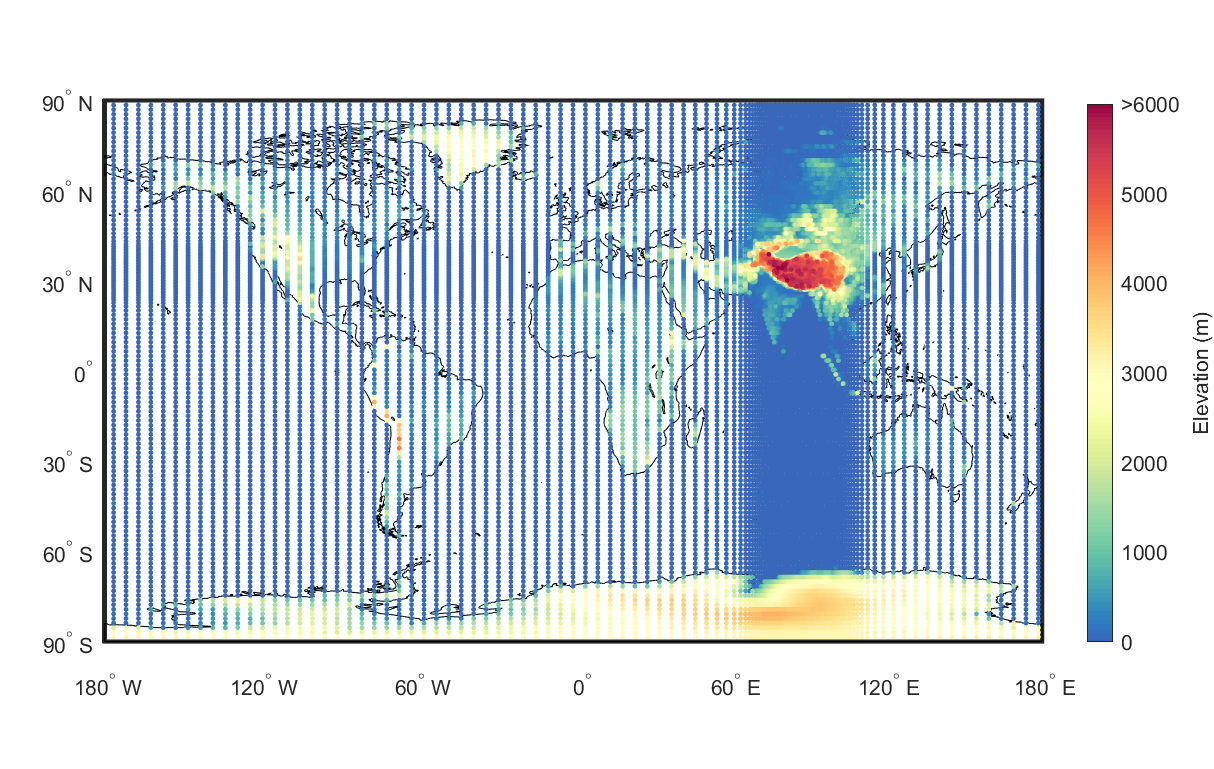


**Supplementary Figure 9. The irregular horizontal grid applied in LMDZOR.** The location of points represents the center latitude and longitude of the grid while the color of points represents the elevation of the grid.

**LMDZOR evaluation**

In this study, we chose five different observation-based and reanalysis datasets to verify the capacity of LMDZOR through the annual and boreal summer hydrological variables and atmospheric circulation. The five datasets include (I) the Climatic Research Unit gridded TS version 4.04 (CRU TS 4.04, <https://crudata.uea.ac.uk/cru/data/hrg/>) ^4^, (II) the gridded gauge-analysis products in Global Precipitation Climatology Center (GPCC, <https://climatedataguide.ucar.edu/climate-data/gpcc-global-precipitation-climatology-centre>) ^5^, (III) the Tropical Rainfall Measuring Mission (TRMM, <https://disc.gsfc.nasa.gov/datasets?keywords=TRMM&page=1>) multi-satellite precipitation analyses ^6^, (IV) the fifth generation ECMWF reanalysis (ERA5, <https://cds.climate.copernicus.eu/cdsapp#!/home>) ^7^, and (V) the NCEP-DOE Reanalysis 2 products (NCEP, <https://psl.noaa.gov/data/gridded/data.ncep.reanalysis2.html>) ^8^. We compared the mean precipitation (in both five datasets) and 850hPa zonal/meridional wind (in ERA5 and NCEP) during 2003-2018 CE against the simulations of CTL in LMDZOR. Results are shown in Supplementary Figures 10 and 11.

LMDZOR shows relatively great capability in simulating the precipitation and 850hPa zonal/meridional wind (Supplementary Figures 10 and 11). Annual precipitation, mainly during the boreal summer (Supplementary Figure 11), is relatively higher in the foothills of the Himalayas, the Western Ghats (WG), the west coast of the Bay of Bengal (BOB) and the Maritime Continent (MC) (Supplementary Figure 10). Both the five datasets and CTL have consistent results. In the boreal summer, ERA5, NCEP, and CTL all show strong westerly winds across the Arabian Sea, the Indian subcontinent, and the BOB (Supplementary Figure 11). These monsoonal winds further turn northward over the South China Sea. Besides, both the datasets and CTL show a strong Western Pacific Subtropical High (WPSH) in the West Pacific. However, CTL in LMDZOR underestimates both the annual and the boreal summer precipitation in the Central Pacific, and the simulated WPSH in CTL is more northerly (Supplementary Figures 10 and 11). The biases of CTL may be due to the lack of an ocean module in the model.


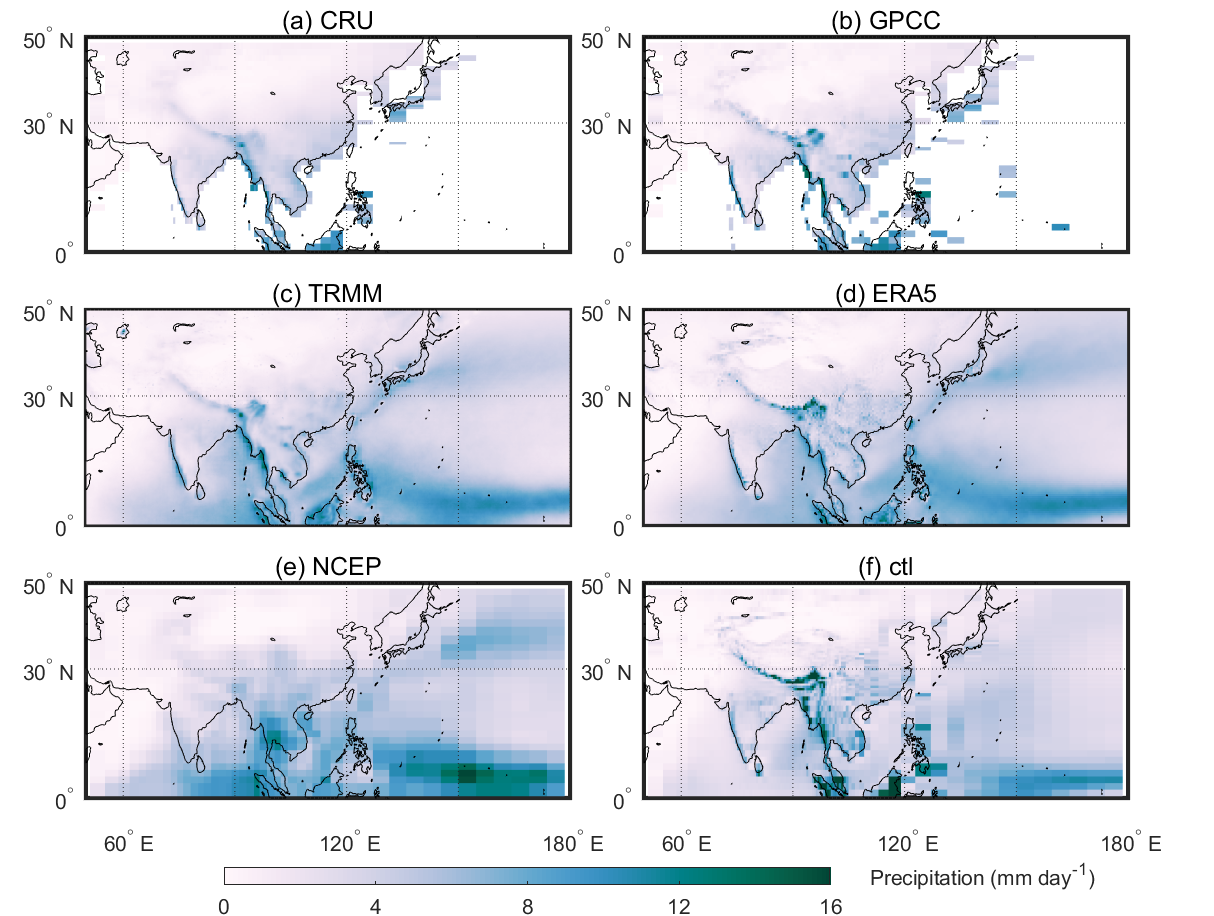


**Supplementary Figure 10.** **Spatial patterns of annual precipitation over eastern Eurasia derived from (a) CRU, (b) GPCC, (c) TRMM, (d) ERA5, (e) NCEP, and (f) CTL in LMDZOR.** Regions without precipitation values in panels (a) and (b) are masked as white.

**
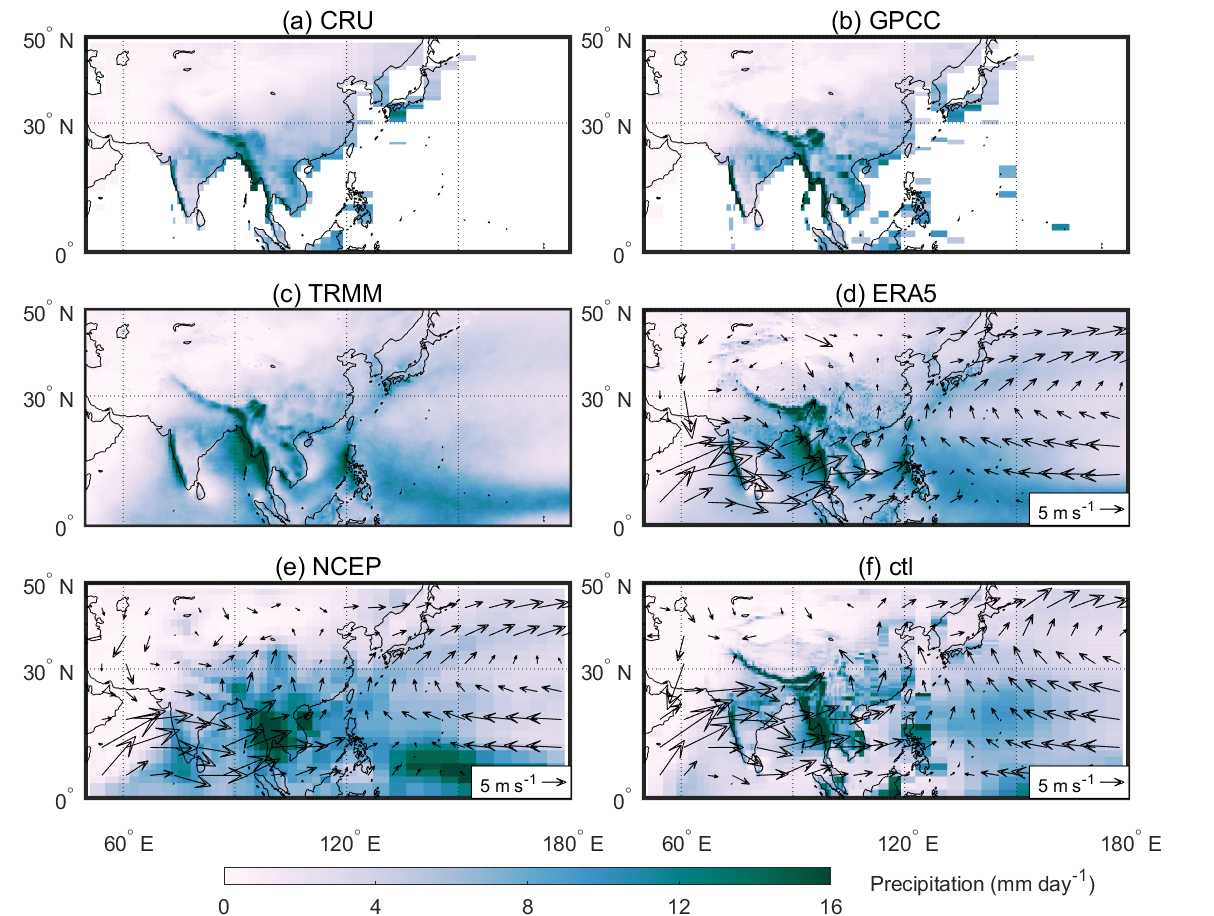
**

**Supplementary Figure 11.** **Spatial patterns of boreal summer precipitation and zonal/meridional wind at 850hPa over eastern Eurasia derived from (a) CRU, (b) GPCC, (c) TRMM, (d) ERA5, (e) NCEP, and (f) CTL in LMDZOR.** Regions without precipitation values in panels (a) and (b) are masked as white.

**OGGM evaluation**

To evaluate the suitability of the OGGM set-up and parameters, we simulated glaciers in the TP and its surrounding ranges over 2000-2016 CE using pre-processed CRU climate data ^9^ (see Methods) instead of the climate data from LMDZOR experiments. The set-up and parameters used in this transient simulation are the same as those of glacier simulations in the manuscript (CTL_t, SCE_t, CTL_e, and SCE_e).

This CRU-based simulation shows that glaciers over the TP and its surrounding ranges undergo a considerable shrinkage during 2000-2016 CE, which is manifested as the loss proportion of glacier area (-1.0%) and glacier volume (-5.2%) (Supplementary Figure 12). Spatially, the glacier retreat over 2000-2016 CE is substantial in the southeast part of the TP, such as the Hengduan Shan (area: -3.1%, volume: -18.1%) and the southeast TP (area: -4.1%, volume: -17.0%), followed by the east Himalaya (area: -2.9%, volume: -12.1%) and the Qilian Shan (area: -2.4%, volume: -10.6%). By contrast, glaciers slightly retreat and even advance in regions such as the east Kun Lun (area: 0.8%, volume: -2.3%), the Karakoram (area: 0.3%, volume: -1.1%), and the west Kun Lun (area: 2.0%, volume: 0.5%) (Supplementary Figure 12). The simulated spatial patterns of glacier changes in OGGM are similar to the previous satellite-based studies, such as ref ^10^ and ref ^11^, which all show shrinking glaciers in the Hengduan Shan and the southeast TP while glaciers in the west Kun Lun and the Karakoram only slightly retreat or even advance. However, compared to the previous studies, OGGM partly underestimates the decrease in glacier area in the southwest part of the TP.

We further compared the glacier thickness changes over the period 2000-2016 CE to those in ref ^12^. Our results show that the mean glacier thickness change over the period 2000-2016 CE is about -0.38 ± 0.25 m yr^-1^, which is slightly lower than that in ref ^12^ (-0.49 ± 0.31 m yr^-1^). For most of the glaciers (88%), the magnitude of the model-data difference is less than 0.45 m yr^-1^ (Supplementary Figure 13).

We also evaluate the performance of OGGM in simulating the area-weighted average equilibrium-line altitude (ELA) of glaciers over 2000-2016 CE (Supplementary Figure 14). Our analysis shows that the area-weighted average ELA in CRU-based simulation over the period 2000-2016 CE is slightly higher than ref ^13^ (90 ± 6 m). Specifically, OGGM overestimates ELA in the west and central part of the TP, such as the west Kun Lun (210 m), the west Himalaya (201 m), and the inner Tibet (142 m) (Supplementary Figure 14). In contrast, OGGM partly underestimates ELA in the southeast part of the TP, including the east Himalaya (-68 m) and the Hengduan Shan (-57 m) (Supplementary Figure 14). In the central Himalay (-5 m) and the northwest part of the TP, such as the Hissar Alay (-21 m) and the Pamir (-20 m), the ELA in CRU-based simulation over the period 2000-2016 CE is generally consistent with that in ref ^13^ (Supplementary Figure 14).

**
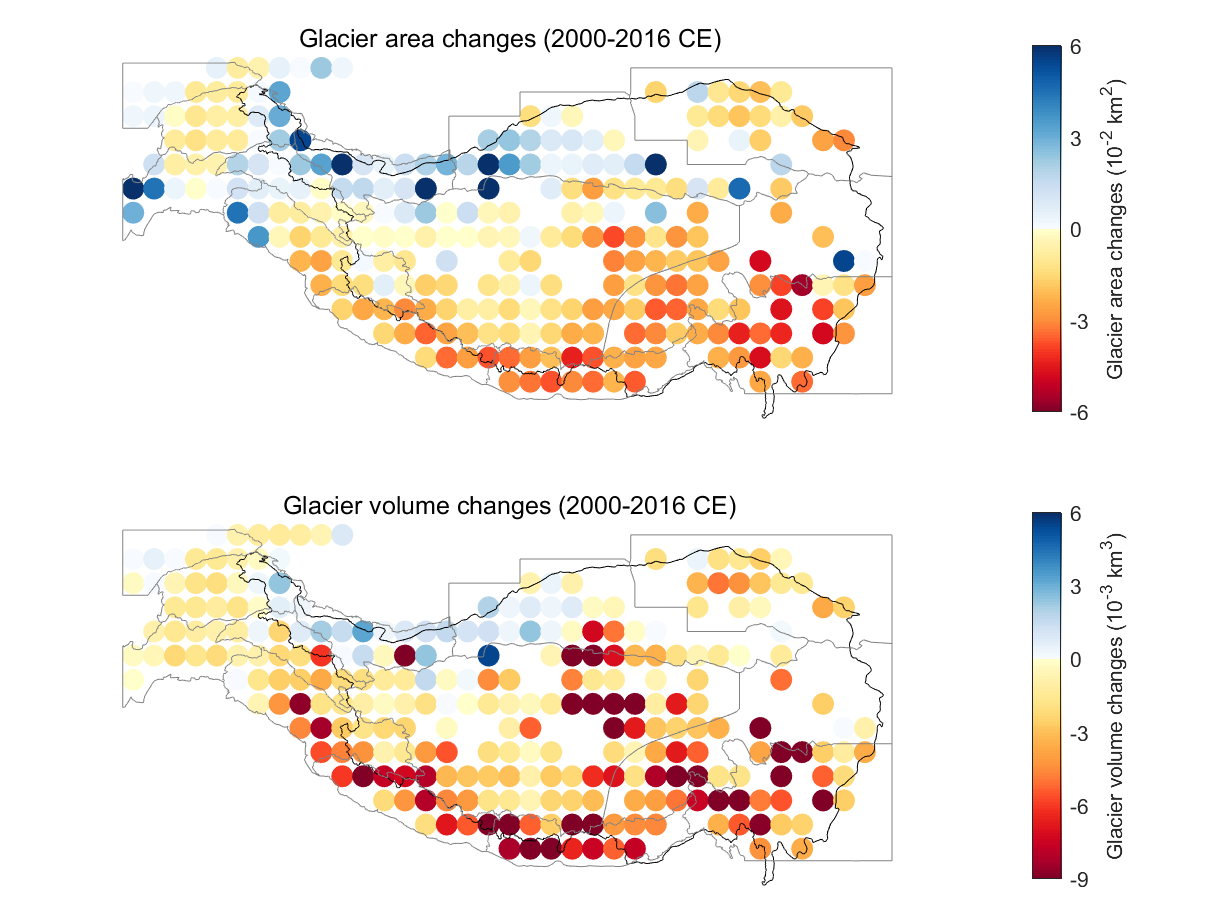
**

**Supplementary Figure 12.** **Spatial patterns of the average changes in glacier (a) area and (b) volume over 2000-2016 CE simulated with OGGM.** The black line indicates the boundary of Tibetan Plateau. The grey lines indicate the boundaries of the 13 second-order regions defined by the RGI v6 ^1, 2^. More details about these regions are shown in Supplementary Table 1. The size of each circle represents the glacier changes over 2000-2016 CE at a spatial resolution of 1°.


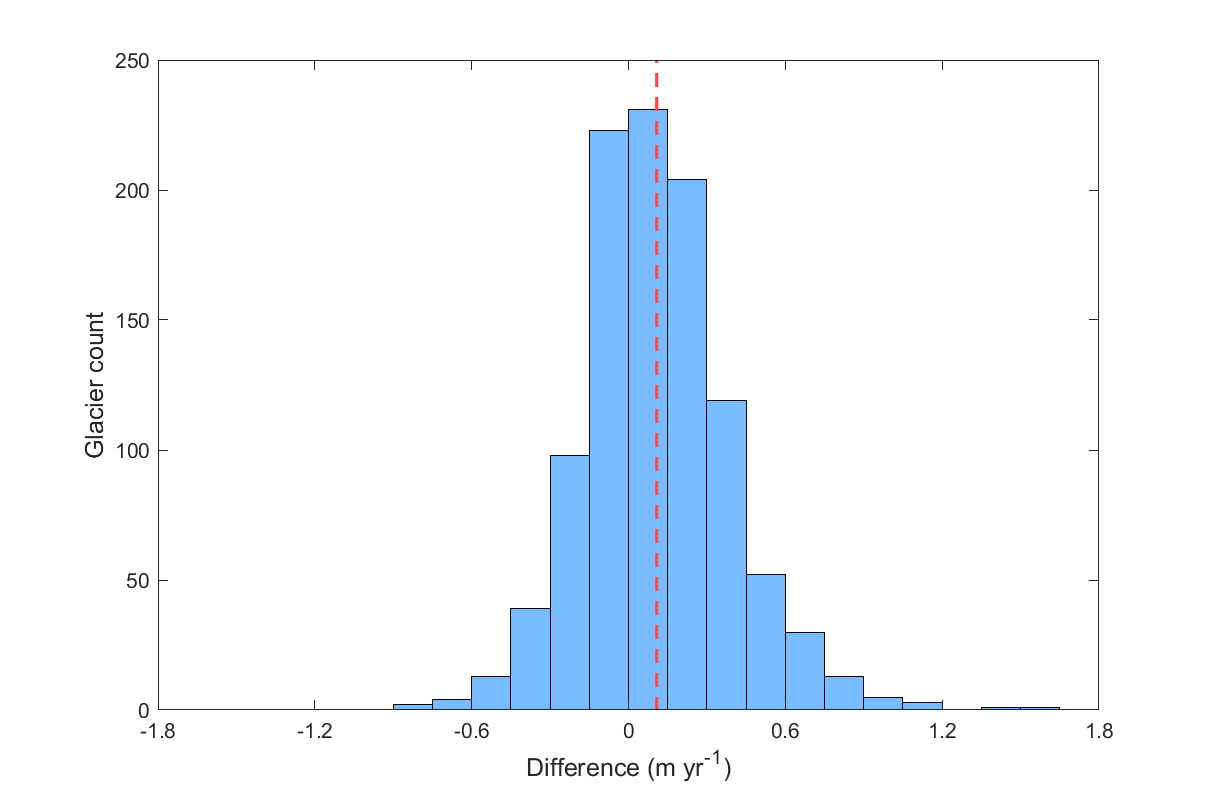


**Supplementary Figure 13. The difference in glacier thickness change over the period 2000-2016 CE between CRU-based simulation and ref ^12^.** The red dashed line is the difference of mean glacier thickness change between CRU-based simulation and ref ^12^.


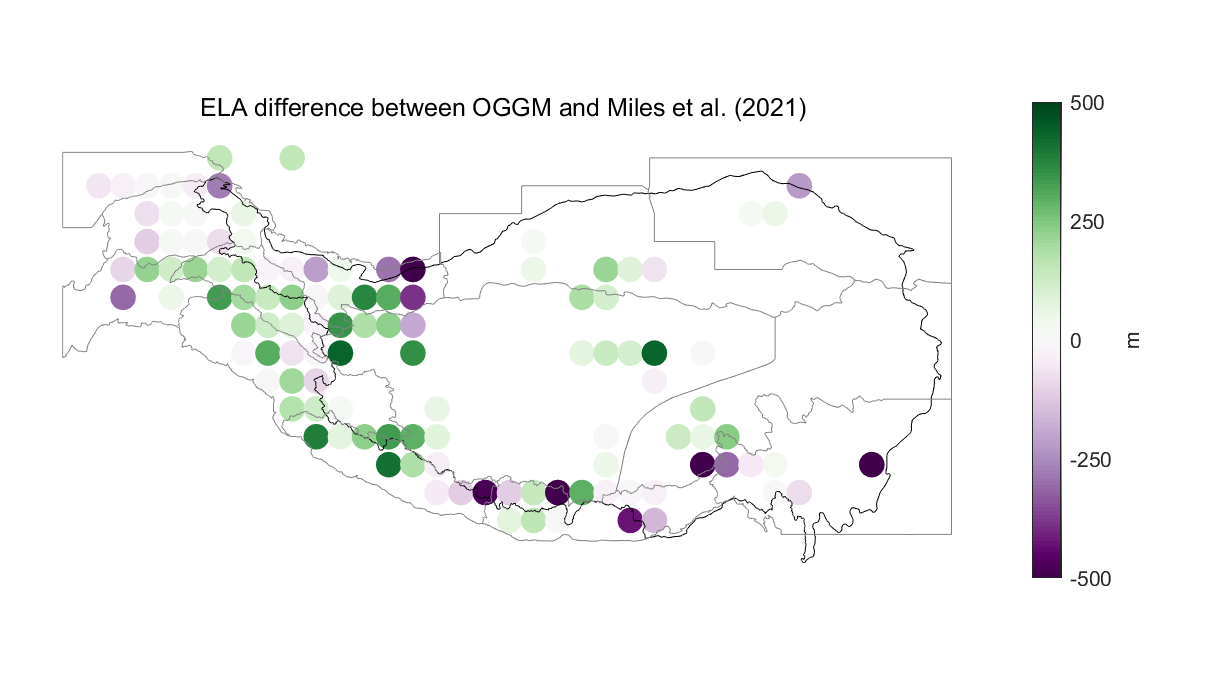


**Supplementary Figure 14. Spatial pattern of the difference of area-weighted average equilibrium-line altitude (ELA) of glaciers between OGGM and ref ^13^ in the Tibetan Plateau (TP) and its surrounding ranges over the period 2000-2016 CE.** The black line indicates the boundary of TP. The grey lines indicate the boundaries of the 13 second-order regions defined by the RGI v6 ^1, 2^. More details about these regions are shown in Supplementary Table 1. The size of each circle represents the ELA difference between OGGM and ref ^13^ at a spatial resolution of 1°.


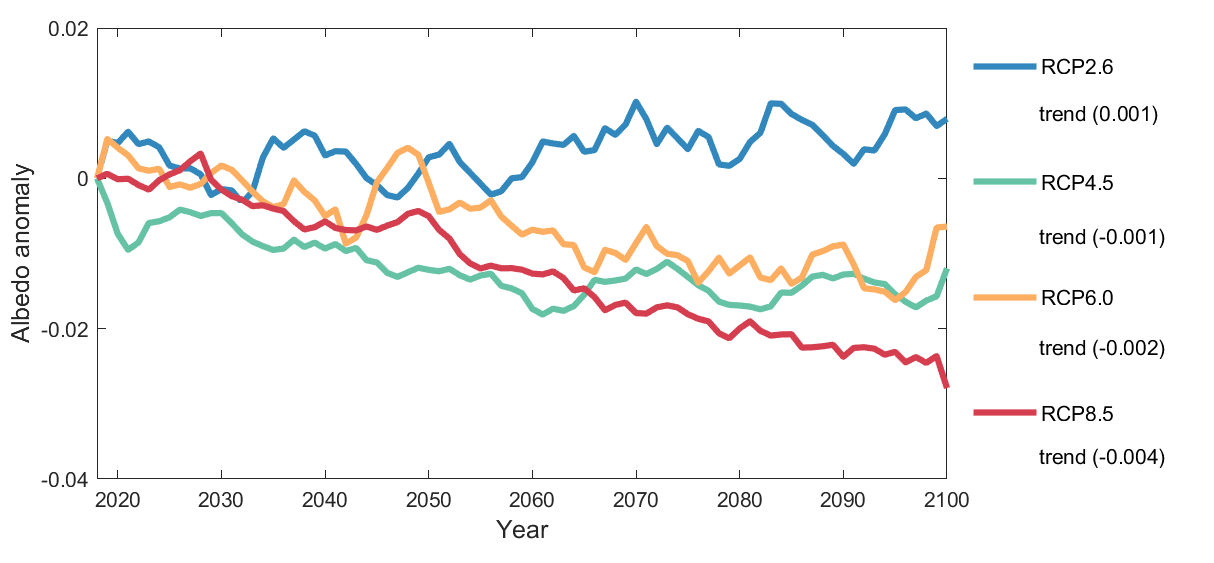


**Supplementary Figure 15. Time series of Tibetan Plateau (TP) surface albedo anomaly projection during 2018-2100 CE**. The albedo anomalies are the 5-year moving average of the annual mean albedo derived from a weighted average of CMIP5 global climate models (GCMs) under different scenarios relative to its benchmark-year (2018 CE) value. The blue, green, orange, and red lines represent the TP surface albedo anomaly projection under the RCP2.6, RCP4.5, RCP6.0, and RCP8.5 scenarios, respectively. The unit of trend is dec^-1^.

**Albedo evaluation**

In this subsection, we utilized the observation to evaluate surface albedo in the TP from a weighted average of 35 eligible CMIP5 GCMs under the historical (2003-2005 CE) and RCP8.5 (2006-2018 CE) scenarios. We used the Bayesian Model Averaging (BMA) to assign model weights for each GCM (see description of BMA in the Methods). Specifically, given albedo projections from GCMs and the observed data from the Moderate Resolution Imaging Spectroradiometer (MODIS) MCD43 product, the weighted predicted probability density functions (PDFs) for albedo projections during 2003-2018 CE are obtained based on the following equation:

$p\left( alb | {alb}_{1},{alb}_{2},\cdots,{alb}_{N} \right)=\sum_{i=1}^{N} w_{i}h_{i}\left( alb | {alb}_{i} \right)$ (1)

where ${alb}_{i}$ is the albedo projection simulated by the $i_{th}$ CMIP5 GCM. $w_{i}$, which is the posterior probability of $i_{th}$ GCM being the best model, is the optimal weight for the $i_{th}$ GCM. $N$ refers to the number of GCMs. $h_{i}\left( alb | {alb}_{i} \right)$ denotes the conditional distribution of albedo projection given that the $i_{th}$ GCM is the best model, and is approximated by a PDF of normal distribution with mean $\left( a_{i}+b_{i}{alb}_{i} \right)$ and standard deviation $\sigma$. The BMA mean and variance are respectively computed as:

$E\left[ \left. alb \right|{alb}_{1},{alb}_{2},\cdots,{alb}_{i} \right]=\sum_{i=1}^{N} w_{i}\left( a_{i}+b_{i}{alb}_{i} \right)$ (2)

$Var\left[ \left. alb \right|{alb}_{1},{alb}_{2},\cdots,{alb}_{i} \right]=\sum_{i=1}^{N} w_{i}\left[ \left( a_{i}+b_{i}{alb}_{i} \right)-\sum_{j=1}^{N} w_{j}\left( a_{j}+b_{j}{alb}_{j} \right) \right]+\sigma^{2}$ (3)

Successful implementation of the BMA method requires estimates of $a_{i}$, $b_{i}$, $w_{i}$ and $\sigma$. The values $a_{i}$ and $b_{i}$ are bias correction terms that are simply estimated from the linear regression of observed albedo on ${alb}_{i}$. Through assuming that model errors are independent across time and space, the log-likelihood function for Equation (1) is:

$L\left( w_{1},w_{2},\cdots,w_{N},\sigma^{2} | {alb}_{1},{alb}_{2},\cdots,{alb}_{N},alb \right)=\sum_{s=1}^{S} log\sum_{i=1}^{N} w_{i}h_{i}\left( alb | {alb}_{i,s} \right)$ (4)

where $S$ is the total number of grid cells over the TP. $h_{i}\left( alb | {alb}_{i,s} \right)$ is the conditional PDF of observed albedo on GCM projection at the $s_{th}$ grid given that the $i_{th}$ GCM is the best model for that grid cell. Since no analytical solutions can be used to maximize this log-likelihood function, we adopted iterative techniques to estimate the maximum likelihood values of weights and variance through a Markov chain Monte Carlo algorithm ^14^.

Our results show that the weighted average of CMIP5 GCMs has only slight biases by about 0.020 (8.6% compared with observation) compared with the observed surface albedo in the TP during the period 2003-2018 CE (Supplementary Figure 16a). Such biases may originate from the overestimation of model projections in the western TP (Supplementary Figure 16b). Moreover, the weighted average of CMIP5 GCMs is also able to correctly capture observed surface albedo changes (model: -0.005 dec^-1^, p<0.05, Mann-Kendall test; observation: -0.006 dec^-1^, p=0.19) (Supplementary Figure 16a). Therefore, the weighted average of CMIP5 GCMs represents surface albedo changes over the TP sufficiently well.

**
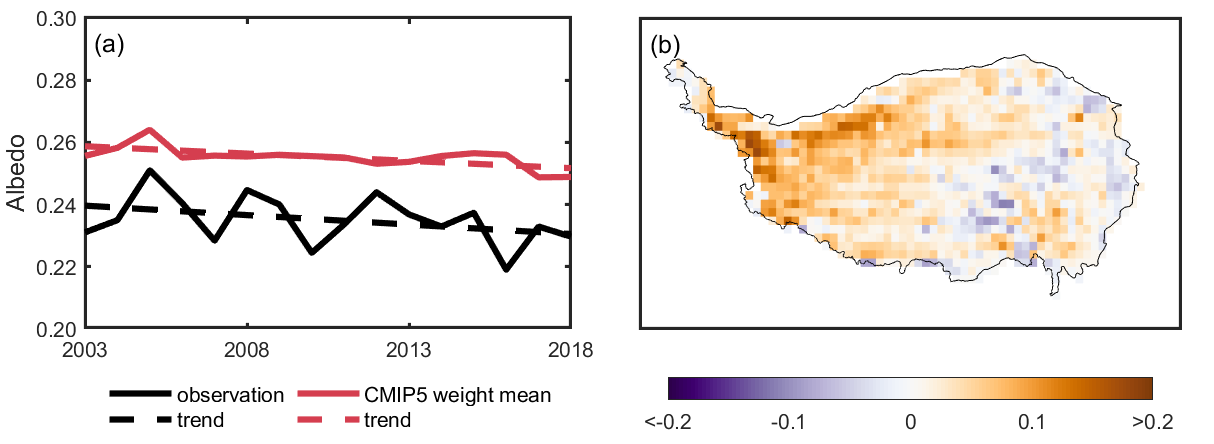
Supplementary Figure 16. Changes of surface albedo in the Tibetan Plateau (TP) and its difference between model projection and observation.** Panel a shows the time series of TP surface albedo over 2003-2018 CE. The thick solid black line represents the observed surface albedo from the MODIS MCD43 product (observation). The thick solid red line is the weighted average of CMIP5 global climate models (GCMs) (CMIP5 weight mean) running under the historical (2003-2005 CE) and RCP8.5 (2006-2018 CE) scenarios. The thick dashed lines are the trend of the corresponding thick solid lines. Panel b indicates the spatial pattern of TP surface albedo difference between CMIP5 weight mean under the historical+RCP8.5 scenario and observation during 2003-2018 CE. The black line indicates the boundary of TP.

**Explanation about glacier volume increasing in glacier simulations**

In the glacier simulations of the manuscript (CTL_t, SCE_t, CTL_e and SCE_e), the sum of glacier volume in the TP and its surrounding ranges slightly increases (2.2% relative to the initial value) in the transient simulation (centennial scale), which further reaches 28.8% (relative to the initial value) in the equilibrium simulation (equilibrium state). Spatially, the increase of glacier volume mainly occurs in the west part of the TP (Supplementary Figure 17), such as the Hindu Kush (transient simulation: 49.4%, equilibrium simulation: 149.1%), the west Kun Lun (9.5%, 82.6%), the east Kun Lun (-4.7%, 68.0%) and the Pamir (13.3%, 43.3%). Since the set-up of OGGM has been evaluated (see OGGM evaluation subsection above), the phenomenon of the increasing glacier volume does not harm our study, which focuses on the difference of glaciers between the control and the scenario simulations. Following is the explanation of the possible reasons behind this phenomenon:

1) Previous studies ^15, 16, 17^ have found that glaciers in the west part of the High Mountain Asia (HMA) exhibited exceptionally little change relative to most of the world’s glaciers in recent years, and this phenomenon is known as the “Karakoram Anomaly”. The suitable climate conditions in recent decades have led to the positive trends of glacier mass balance over this region, which may contribute to the “Karakoram Anomaly” ^18^. Since the boundary conditions in LMDZOR experiments are derived from the products during 2003-2018 CE (see Methods) and the climate output of these experiments is used to drive OGGM, if the climate over this period is also suitable for advancing glaciers, the “Karakoram Anomaly” may exist in our simulations as well. Precipitation over this period (2003-2018 CE) shows an increased anomaly in some regions of the west part of the TP (Hindu Kush: 1.82 mm month^-1^; west Kun Lun: 0.46 mm month^-1^, east Kun Lun: 0.60 mm month^-1^, Pamir: 1.37 mm month^-1^) compared with the climatology condition (Supplementary Figure 18). Therefore, it is not surprising that about 93.1% (81.7%) of these glaciers with increased volume in CRU simulation (simulated glaciers over the period 2000-2016 CE, see OGGM evaluation subsection above) will continue to increase in the transient (equilibrium) simulations (Supplementary Table 2). Glacier volume increases in glacier simulations may be partly due to the “Karakoram Anomaly”.

2) During the pre-processing procedure, the difference or ratio between the first 16-year climate data in LMDZOR and the pre-processed CRU climate data over the reference climate period is first computed. Then, the deltas (difference or ratio) between two climate data are applied to the rest 83-year results of LMDZOR to obtain the climate forcing of OGGM glacier simulations (see Methods). Compared with the first 16-year climate data in LMDZOR, the final 83-year temperature in the west part of the TP remains stable (Hindu Kush: 0.02 K; west Kun Lun: 0.03 K, east Kun Lun: 0.05 K, Pamir: 0.03 K), while the final 83-year precipitation in the west part of the TP is much higher (Hindu Kush: 1.04 mm month^-1^; west Kun Lun: 0.44 mm month^-1^, east Kun Lun: 0.18 mm month^-1^, Pamir: 0.86 mm month^-1^) (Supplementary Figure 19). Therefore, the final 83-year climate in LMDZOR is more suitable for glaciers in the west part of the TP compared with the climate of LMDZOR in the first 16 years. The latter point may explain the rest part of glaciers with increased volume in glacier simulations.

**
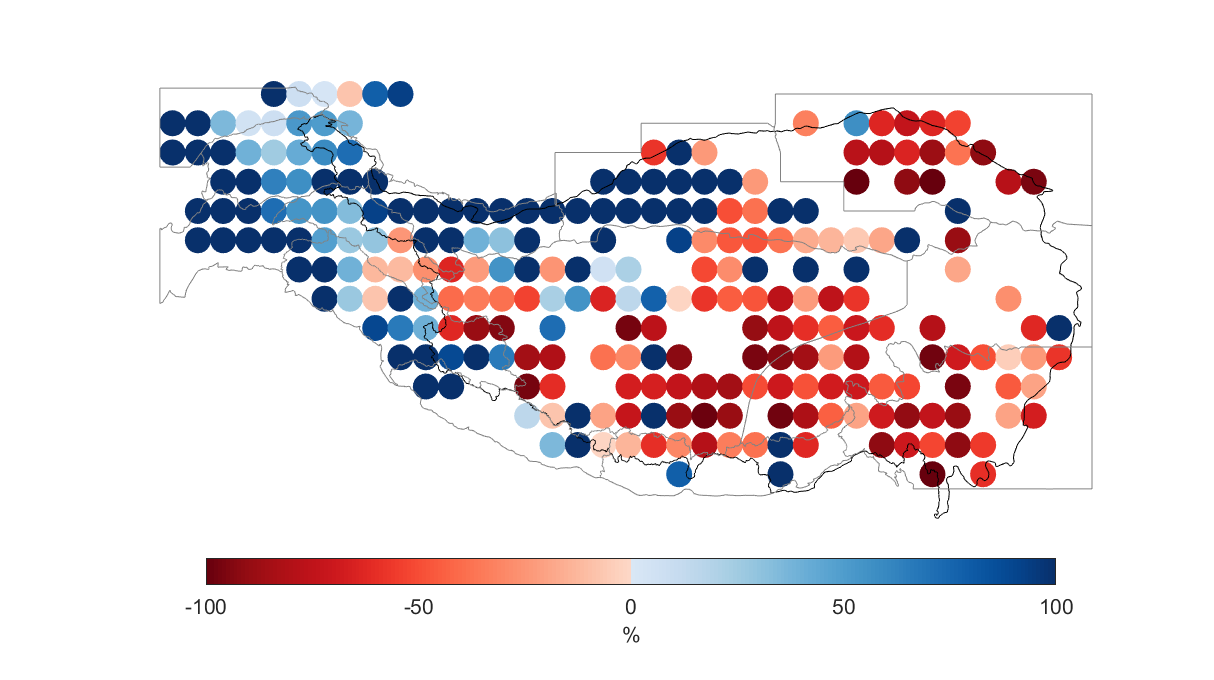
**

**Supplementary Figure 17. The spatial pattern of the proportion of glacier volume difference between the equilibrium-state and initial value.** The proportion here indicates the glacier volume of CTL_e averaged over the final 1000 years divided by that in the initial state. The black line indicates the boundary of Tibetan Plateau. The grey lines indicate the boundaries of the 13 second-order regions defined by the RGI v6 ^1, 2^. More details about these regions are shown in Supplementary Table 1. The size of each circle represents the proportion of glacier volume difference at a spatial resolution of 1°.


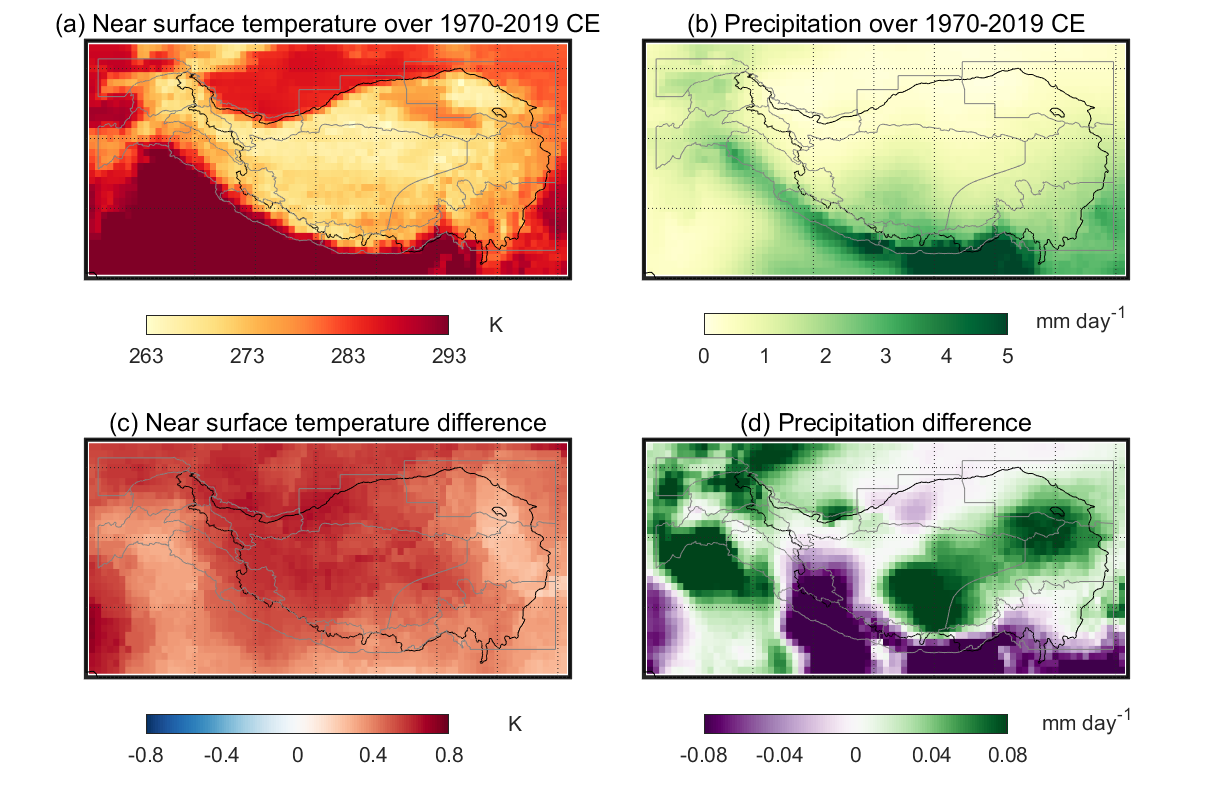
**Supplementary Figure 18. Spatial patterns of the climate over the period 1970-2019 CE (panels a and b), and its difference to the climate over the period 2003-2018 CE (panels c and d).** The climate data, including near-surface air temperature (panels a and c) and precipitation (panels b and d), is derived from CRU TS. The climate difference between two time periods in the lower row is computed as the difference between the climate over the period 2003-2018 CE and that over the period 1970-2019 CE. The black line indicates the boundary of Tibetan Plateau. The grey lines indicate the boundaries of the 13 second-order regions defined by the RGI v6 ^1, 2^. More details about these regions are shown in Supplementary Table 1.

**Supplementary Table 2. Total number of glaciers with increased volume and its proportion to the total number of glaciers over the Tibetan Plateau (TP) and its surrounding ranges in different glacier simulations.** The upper table indicates the respective glaciers’ volume increases only in one glacier simulation, while the lower table indicates the respective glaciers’ volume increases in both glacier simulations.

| CRU simulation | CTL_t | CTL_e |
| --- | --- | --- |
| 25,591 (25.4%) | 44,571 (44.3%) | 42,412 (42.1%) |
| CRU simulation && CTL_t | CRU simulation && CTL_e | CTL_t && CTL_e |
| 23,826 (23.7%) | 20,907 (20.8%) | 21,505 (21.4%) |


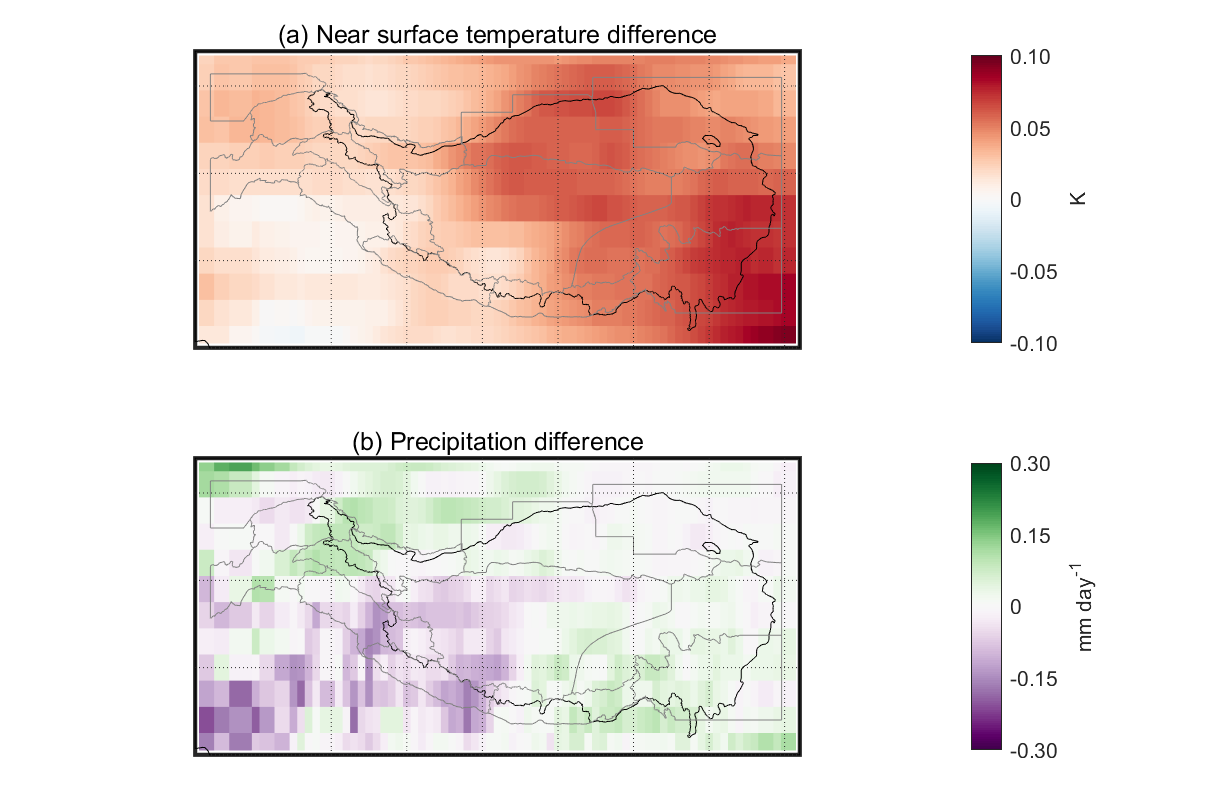


**Supplementary Figure 19. Spatial patterns of the difference between the final 83-year and first 16-year climate in LMDZOR experiments.** The climate data, including (a) near-surface air temperature and (b) precipitation, is derived from CTL in LMDZOR. The black line indicates the boundary of Tibetan Plateau. The grey lines indicate the boundaries of the 13 second-order regions defined by the RGI v6 ^1, 2^. More details about these regions are shown in Supplementary Table 1.

**Supplementary Reference**

1. Pfeffer WT, et al. The Randolph Glacier inventory: a globally complete inventory of glaciers. *J Glaciol* **60**, 537-552 (2014).

2. RGI Consortium. Randolph Glacier Inventory – A Dataset of Global Glacier Outlines: Version 6.0: Technical Report. (ed Global Land Ice Measurements from Space C, USA. Digital Media.) (2017).

3. Farinotti D, et al. A consensus estimate for the ice thickness distribution of all glaciers on Earth. *Nat Geosci* **12**, 168-+ (2019).

4. Harris I, Osborn TJ, Jones P, Lister D. Version 4 of the CRU TS monthly high-resolution gridded multivariate climate dataset. *Sci Data* **7**, 109 (2020).

5. Schneider U, et al. GPCC's new land surface precipitation climatology based on quality-controlled in situ data and its role in quantifying the global water cycle. *Theor Appl Climatol* **115**, 15-40 (2014).

6. Kummerow, et al. The Status of the Tropical Rainfall Measuring Mission (TRMM) after Two Years in Orbit. *J Appl Meteorol* **39**, 1965-1982 (2000).

7. Hersbach H, Bell, B., Berrisford, P., Biavati, G., Horányi, A., Muñoz Sabater, J., Nicolas, J., Peubey, C., Radu, R., Rozum, I., Schepers, D., Simmons, A., Soci, C., Dee, D., Thépaut, J-N. ERA5 monthly averaged data on pressure levels from 1979 to present. (ed (CDS) CCCSCSCDS) (2019).

8. Kanamitsu M, et al. NCEP–DOE AMIP-II Reanalysis (R-2). *Bull Am Meteorol Soc* **83**, 1631-1644 (2002).

9. Maussion F, et al. The Open Global Glacier Model (OGGM) v1.1. *Geosci Model Dev* **12**, 909-931 (2019).

10. Brun F, Berthier E, Wagnon P, Kaab A, Treichler D. A spatially resolved estimate of High Mountain Asia glacier mass balances from 2000 to 2016. *Nat Geosci* **10**, 668-673 (2017).

11. Dehecq A, et al. Twenty-first century glacier slowdown driven by mass loss in High Mountain Asia. *Nat Geosci* **12**, 22-27 (2019).

12. Maurer JM, Schaefer JM, Rupper S, Corley A. Acceleration of ice loss across the Himalayas over the past 40 years. *Sci Adv* **5**, eaav7266 (2019).

13. Miles E, et al. Health and sustainability of glaciers in High Mountain Asia. *Nat Commun* **12**, 2868-2868 (2021).

14. Vrugt J, Diks C, Clark M. Ensemble Bayesian model averaging using Markov Chain Monte Carlo sampling. *Environmental Fluid Mechanics* **8**, 579-595 (2008).

15. Yao T, et al. Different glacier status with atmospheric circulations in Tibetan Plateau and surroundings. *Nat Clim Chang* **2**, 663-667 (2012).

16. Azam MF, et al. Glaciohydrology of the Himalaya-Karakoram. *Science* **373**, eabf3668 (2021).

17. Farinotti D, Immerzeel WW, De Kok RJ, Quincey DJ, Dehecq A. Manifestations and mechanisms of the Karakoram glacier Anomaly. *Nat Geosci* **13**, 8-16 (2020).

18. Hewitt K. The Karakoram Anomaly? Glacier Expansion and the ‘Elevation Effect,’ Karakoram Himalaya. *Mt Res Dev* **25**, 332-340, 339 (2005).
